# Localization and Classification of Adrenal Masses in Multiphase Computed Tomography: Retrospective Study

**DOI:** 10.2196/65937

**Published:** 2025-04-24

**Authors:** Liuyang Yang, Xinzhang Zhang, Zhenhui Li, Jian Wang, Yiwen Zhang, Liyu Shan, Xin Shi, Yapeng Si, Shuailong Wang, Lin Li, Ping Wu, Ning Xu, Lizhu Liu, Junfeng Yang, Jinjun Leng, Maolin Yang, Zhuorui Zhang, Junfeng Wang, Xingxiang Dong, Guangjun Yang, Ruiying Yan, Wei Li, Zhimin Liu, Wenliang Li

**Affiliations:** 1 Yunnan Cancer Hospital The Third Affiliated Hospital of Kunming Medical University Kunming China; 2 School of Data Science Fudan University Shanghai China; 3 The Affiliated Hospital of Kunming University of Science and Technology Kunming China; 4 Department of Urology The First People's Hospital of Yunnan Province Kunming China; 5 Medical School Kunming University of Science and Technology Kunming China; 6 Department of Urology Second Affiliated Hospital of Kunming Medical University Kunming China; 7 Department of Urology Honghe Autonomous Prefecture 3rd Hospital Kunming China; 8 Kunming Medical University Kunming China

**Keywords:** MA-YOLO model, multi-class adrenal masses, multi-phase CT images, localization, classification

## Abstract

**Background:**

The incidence of adrenal incidentalomas is increasing annually, and most types of adrenal masses require surgical intervention. Accurate classification of common adrenal masses based on tumor computed tomography (CT) images by radiologists or clinicians requires extensive experience and is often challenging, which increases the workload of radiologists and leads to unnecessary adrenal surgeries. There is an urgent need for a fully automated, noninvasive, and precise approach for the identification and accurate classification of common adrenal masses.

**Objective:**

This study aims to enhance diagnostic efficiency and transform the current clinical practice of preoperative diagnosis of adrenal masses.

**Methods:**

This study is a retrospective analysis that includes patients with adrenal masses who underwent adrenalectomy from January 1, 2021, to May 31, 2023, at Center 1 (internal dataset), and from January 1, 2016, to May 31, 2023, at Center 2 (external dataset). The images include unenhanced, arterial, and venous phases, with 21,649 images used for the training set, 2406 images used for the validation set, and 12,857 images used for the external test set. We invited 3 experienced radiologists to precisely annotate the images, and these annotations served as references. We developed a deep learning–based adrenal mass detection model, Multi-Attention YOLO (MA-YOLO), which can automatically localize and classify 6 common types of adrenal masses. In order to scientifically evaluate the model performance, we used a variety of evaluation metrics, in addition, we compared the improvement in diagnostic efficacy of 6 doctors after incorporating model assistance.

**Results:**

A total of 516 patients were included. In the external test set, the MA-YOLO model achieved an intersection over union of 0.838, 0.885, and 0.890 for the localization of 6 types of adrenal masses in unenhanced, arterial, and venous phase CT images, respectively. The corresponding mean average precision for classification was 0.885, 0.913, and 0.915, respectively. Additionally, with the assistance of this model, the classification diagnostic performance of 6 radiologists and clinicians for adrenal masses improved. Except for adrenal cysts, at least 1 physician significantly improved diagnostic performance for the other 5 types of tumors. Notably, in the categories of adrenal adenoma (for senior clinician: *P*=.04, junior radiologist: *P*=.01, and senior radiologist: *P*=.01) and adrenal cortical carcinoma (junior clinician: *P*=.02, junior radiologist: *P*=.01, and intermediate radiologist: *P*=.001), half of the physicians showed significant improvements after using the model for assistance.

**Conclusions:**

The MA-YOLO model demonstrates the ability to achieve efficient, accurate, and noninvasive preoperative localization and classification of common adrenal masses in CT examinations, showing promising potential for future applications.

## Introduction

Adrenal incidentaloma refers to an adrenal mass incidentally discovered during nonadrenal imaging studies, such as abdominal computed tomography (CT) or magnetic resonance imaging. These masses are typically nonfunctional, meaning that they do not cause abnormal hormone levels in the body. Although most adrenal incidentalomas are benign, they still require thorough evaluation to exclude possible malignant tumors. Adrenal incidentalomas themselves may not pose a direct threat to health, but some may secrete hormones, leading to hormonal imbalances. For example, some incidentalomas may secrete adrenaline or noradrenaline, which can cause hypertension or other hormone-related symptoms. Additionally, if the tumor is malignant, it may have more serious implications for the health [1,2].

The incidence and detection rate of adrenal incidentalomas have been increasing due to the widespread use of medical imaging and enhanced awareness of health checkups. A retrospective cohort study [3] showed that from 1995 to 2017, the incidence of adrenal tumors increased by approximately 10 times. Regarding detection rate, a study published in 1985 [4] found that 1.3% of individuals undergoing CT scans in the general population were incidentally found to have adrenal nodules, while this rate has recently increased to 5% [5]. The etiology of adrenal incidentalomas can be broadly classified into five categories: (1) adrenal cortical adenoma or nodular hyperplasia, (2) other benign lesions (myelolipoma, cysts, etc), (3) adrenal cortical carcinoma, (4) other malignant tumors (metastatic carcinoma, lymphoma, etc), and (5) pheochromocytoma [6].

Different types of adrenal masses require different management strategies. Generally, definitive classification of adrenal masses relies on pathologic testing, and invasive pathologic testing usually has a negative physical and psychological impact on the patient. Therefore, for adrenal masses, rapid and accurate localization and identification through noninvasive methods are of great significance for disease management. Contrast-enhanced CT, as a noninvasive examination, can provide important references for the classification of adrenal masses [7]. However, locating and classifying adrenal masses based on CT images often requires rich clinical experience and practice in this field, and the accuracy of judgment may vary due to different doctors’ experiences [8], posing significant challenges for clinicians and radiologists.

Currently, deep learning is increasingly being applied in the medical field, particularly showing promising performance in the recognition, segmentation, and partial disease classification of abdominal organs and lesions [9-14]. Deep learning models possess strong feature learning capabilities, allowing them to automatically learn high-level abstract feature representations from data. In medical imaging, the localization and classification of abdominal organs often require consideration of various features such as morphology, texture, and spatial location. Deep learning models can effectively learn these features and demonstrate good generalization performance when dealing with diverse imaging data. Although some studies have used machine learning techniques to focus on the classification and diagnosis of certain types of adrenal masses based on CT [15-19], research on the automatic detection of adrenal masses combined with multiclassification diagnosis using CT remains a largely unexplored area.

Therefore, this study proposes a novel preoperative diagnostic method for adrenal masses—Multi-Attention YOLO (MA-YOLO). Through experimental evaluation, the effectiveness of the MA-YOLO model in the localization and classification of adrenal masses was evaluated, and its potential application in clinical decision support was validated, aiming to improve diagnostic efficiency and accuracy through a noninvasive approach.

## Methods

### Ethical Considerations

This retrospective study was conducted in accordance with the World Medical Association Declaration of Helsinki. It was approved by the Research Ethics Committee of the First People’s Hospital of Yunnan Province (number KHLL2023-KY170) and the Second Affiliated Hospital of Kunming Medical University (number 2023-233). Due to the retrospective and anonymous nature of the analysis, the Ethics Committee waived the need for informed consent. Additionally, all retrospective datasets were deidentified prior to collection to remove any patient-related information. None of the authors were involved in the data deidentification process.

### Study Design

**Figure 1:**
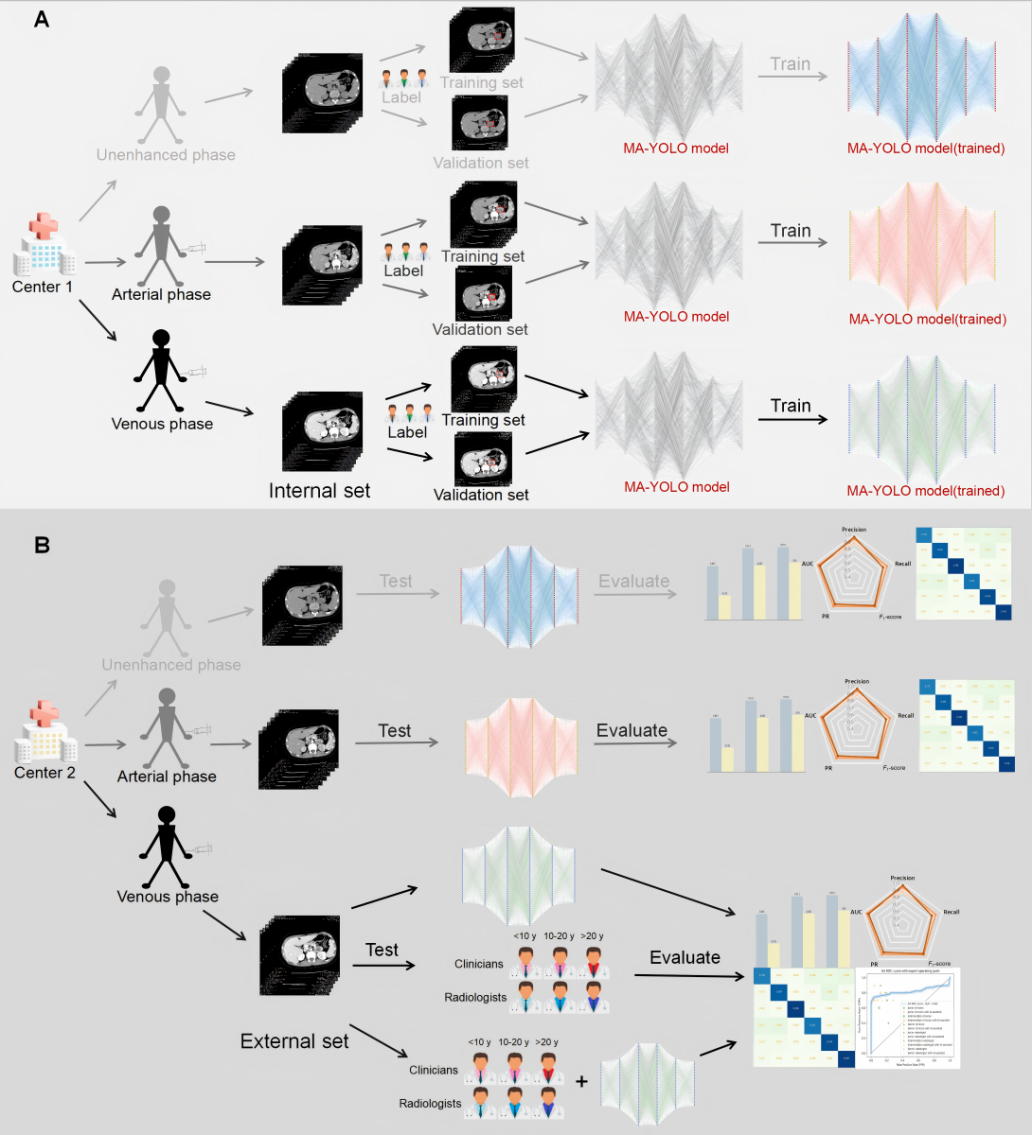
The flowchart of the research. (A) Training and validation of MA-YOLO model on internal sets. (B) Test of MA-YOLO model on external sets, for the venous phase model with the best results, and a model versus physician assisted by model comparison test was used. MA-YOLO: Multi-Attention YOLO.

The flowchart of the study is shown in Figure 1. Our study is divided into 4 main steps, the first step is to create a multicenter large CT image database of adrenal masses, in which the mass types include 6 common adrenal masses, and the CT types include unenhanced, arterial, and venous phases. Second, 3 physicians were invited to provide high-quality annotations of the mass types and locations. Then the MA-YOLO model proposed in this study was used to train the data and achieve accurate localization and classification of 6 types of adrenal masses. Finally, in order to scientifically evaluate the effect of the model proposed in this study, we used the model as well as invited 6 junior, intermediate, and senior clinicians and radiologists, respectively, on an external test dataset and evaluated the diagnostic efficacy of the physicians before and after MA-YOLO assistance, and used decision curve analysis to evaluate the clinical net benefit.

### Data Collection

Our dataset consists of 2 patient cohorts, including patients who underwent adrenalectomy for adrenal masses with clear pathological diagnoses at the Second Affiliated Hospital of Kunming Medical University (internal dataset) from January 1, 2021, to May 31, 2023, and at the First People’s Hospital of Yunnan Province (external dataset) from January 1, 2016, to May 31, 2023. Based on these criteria, a total of 669 and 348 patients were respectively retrieved. Exclusion criteria were defined as follows: (1) incomplete clinical or imaging data, (2) poor image quality due to factors such as respiratory motion, making it difficult to visualize the adrenal region, (3) lesion diameter <1 cm, (4) presence of other lesions within the same cross-sectional slice of the adrenal mass or multiple adrenal masses, and (5) pathological classifications not relevant to this study. According to the aforementioned inclusion and exclusion criteria, a total of 343 and 173 patients were respectively enrolled from the 2 centers (Figure S1 in Multimedia Appendix 1 [20,21]). The dataset included six categories: (1) adrenocortical adenoma (AA; n=249/n=111), (2) pheochromocytoma (n=35/n=38), (3) adrenal myelolipoma (AM; n=19/n=7), (4) adrenal cyst (AC) (n=22/n=8), (5) adrenal ganglioneuroma (AGN; n=11/n=5), and (6) adrenocortical carcinoma (ACC; n=7/n=4). Among all patient images, only those containing adrenal masses were included, totaling 36,911 images, with 12,089, 12,329, and 12,493 images acquired during the unenhanced, arterial, and venous phases, respectively. The patient images from the internal dataset were selected as the training and validation sets in a ratio of 9:1. The patient images from the external dataset were used as the external test set.

This study used various multislice CT scanners (Brilliance iCT, Philips; SOMATOM Force, SOMATOM Drive, or SOMATOM Perspective, Siemens; Aquilion ONE, Canon) for abdominal contrast-enhanced scanning. The collimation settings were 128×0.625, 192×0.6, 128×0.6, 64×0.6, and 100×0.5, with a tube voltage range of 80-120 kVp and a matrix size of 512×512. The gantry rotation time was 0.5 seconds, with a pitch range of 0.5-0.993 and a slice thickness varying between 0.9 mm and 5 mm. For Brilliance iCT, the tube current was set at 200-250 mAs, while for the other scanners, it was automatically adjusted. Nonionic contrast agents, Iohexol Injection or Iomeprol Injection, were administered via the cubital vein at a dosage of 450 mg I/kg, with concentrations of 350 mg I/mL for Iohexol and 400 mg I/mL for Iomeprol. Arterial phase imaging used threshold-triggering technology, while venous phase imaging used a fixed delay of 50-70 seconds.

### Image Annotation

Our annotations consist of class labels for each type of tumor and bounding boxes with localization. This study included 6 common types of adrenal tumors, namely AA, pheochromocytoma (PCC), AM, AC, AGN, and ACC. This bounding box ideally represents the minimum outer rectangle encompassing the entire tumor. Standardization of annotations is crucial for ensuring high-quality annotations; therefore, we implemented a standardized and rigorous image annotation process. All CT images of included patients were directly exported in JPG format from the picture archiving and communication system, with window width and level fixed to commonly used ranges facilitating the identification of adrenal masses (window width: 250-300 Hounsfield units [HU], window level: 30-50 HU).

During annotation, all radiologists were blinded to clinical history and patient information. In the internal dataset, 100 images were randomly selected from each of the 6 categories, totaling 600 adrenal mass images. These images were annotated manually using the LabelImg software (version 1.8.6) by 3 radiologists: 1 radiologist with 17 years of clinical experience, 1 radiologist with 16 years of urological imaging experience, and 1 radiologist with 21 years of imaging experience. Annotations included bounding box selection of the images and classification based on pathological results. Efforts were made to ensure that the bounding boxes fully encompassed the masses. Interlabeler average intersection over union (IoU) was calculated.

After a 1-month clarification period, the same procedure was repeated to calculate the average IoU among labelers. The calculated values for both interlabeler and intralabeler average IoU were found to be greater than 0.90 (Table 1), indicating excellent interlabeler and intralabeler consistency [22]. Subsequently, all images were manually annotated by the aforementioned 3 physicians in a random manner. To ensure accurate labeling of adrenal masses within each image, after 1 physician completed the labeling, the other 2 physicians examined each mark individually. In cases of disagreement among the 3 physicians, a final decision was made collectively after consultation.

**Table 1:** Comparison intersection over union (IoU) between interlabeler and intralabeler.

Method			Average IoU
**Interlabeler comparison**			
	Labeler 1 vs Labeler 1	0.920	
	Labeler 2 vs Labeler 2	0.917	
	Labeler 3 vs Labeler 3	0.917	
**Intralabeler comparison**			
	Labeler 1 vs Labeler 2	0.923	
	Labeler 2 vs Labeler 3	0.922	
	Labeler 1 vs Labeler 3	0.945	

### Development of the MA-YOLO Model

**Figure 2:**
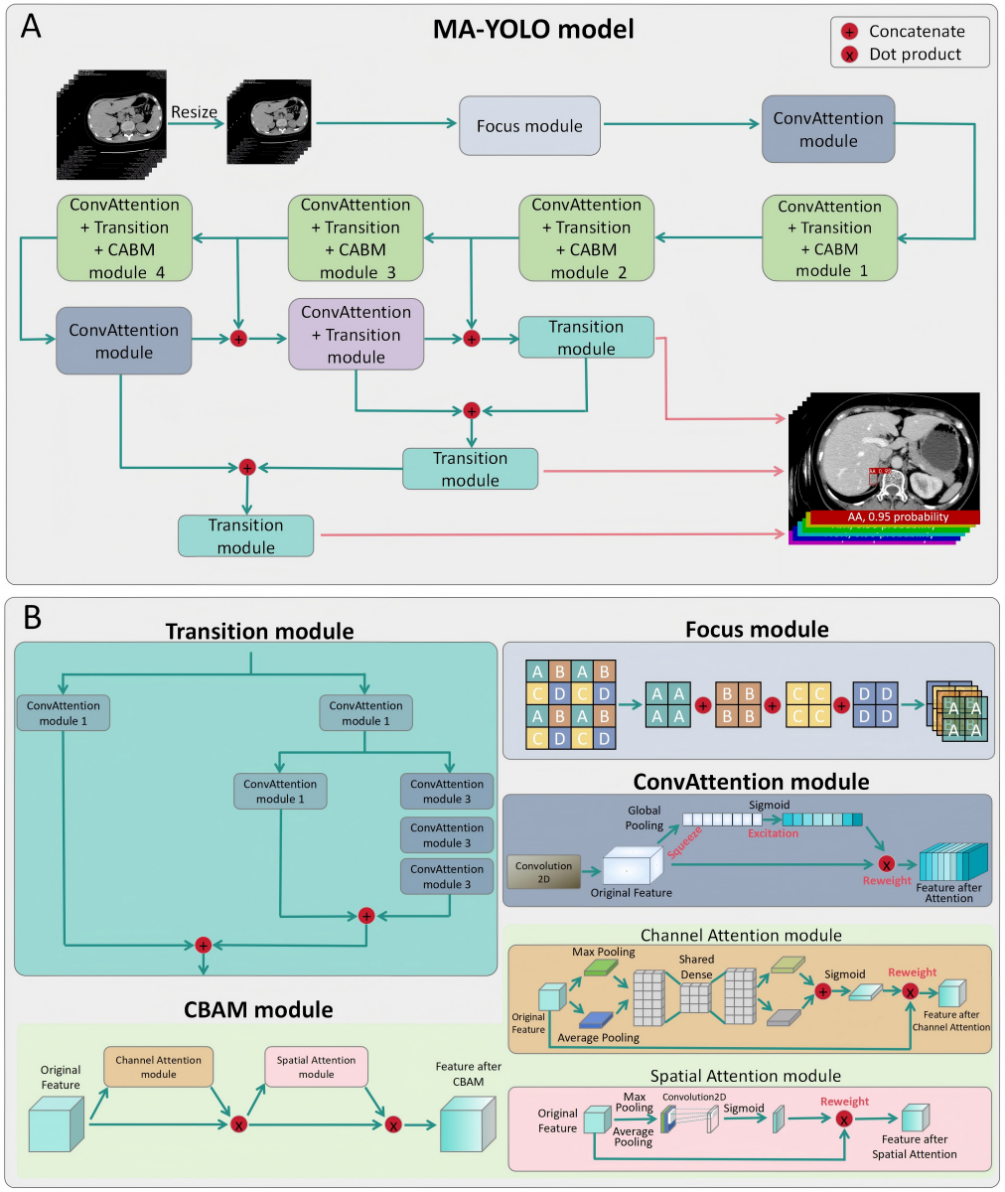
Structure of MA-YOLO model. (A) General structure of the MA-YOLO model. (B) Specific structure of Transition module, Focus module, Convolutional Block Attention module (CBAM), and ConvAttention Module in MA-YOLO model. MA-YOLO: Multi-Attention YOLO.

The model architecture (Figure 2), parameter settings (Table S1 in Multimedia Appendix 1 [20,21]), and other details of the MA-YOLO model can be found in the Multimedia Appendix 1 [20,21].

### Comprehensive Evaluation of the MA-YOLO Model

We initially conducted comparative experiments between the MA-YOLO model and 5 other models, including YOLO V3, V5, and V7, which represent different stages of YOLO development, as well as 2 widely recognized object detection models, faster region-based convolutional neural network (R-CNN) and EfficientDet. We used evaluation indexes such as IoU, mean average precision (mAP), Precision, Recall, and *F*_1_-score to compare and evaluate the model performance, Meanwhile, a decision curve analysis was performed to evaluate the clinical net benefit of the MA-YOLO model.

Subsequently, we compared the MA-YOLO model with physicians. It was achieved by evaluating the potential clinical utility of the constructed classification model by comparing its diagnostic performance with that of physicians independently, and by assisting physicians with model results and comparing them with physicians’ independent diagnoses. By randomly selecting 10 patients from each of the 6 categories within the external test dataset, we chose 1 image each from the unenhanced, arterial, and venous phases of the tumor’s maximum diameter. Six physicians were invited, 3 junior, intermediate, and senior radiologists practicing urologic imaging (with 5, 16, and 21 years of diagnostic imaging experience), and urologic clinicians (with 7, 14, and 27 years of clinical experience). Six physicians were first invited to make a full-blind judgment, then the order of the images was disrupted and a second judgment was made with the assistance of the MA-YOLO model results. Finally, Delong test was used to compare the difference between the physicians’ independent diagnosis and the MA-YOLO model diagnosis results, as well as the difference between the physicians’ independent diagnosis and the diagnosis results assisted by the MA-YOLO model.

### Statistical Analysis

Statistical analysis of patient baseline data was performed using SPSS (version 26.0; IBM Corp). IoU was used to assess the consistency between the model and radiologists in identifying masses. To evaluate the performance of the classification model, we conducted a receiver operating characteristic (ROC) analysis and calculated the area under the receiver operating characteristic curve (AUC). Additionally, Precision, Recall, mAP, Confusion Matrix, and F1-score were used as performance metrics for the classification model. The Delong test was used to compare the differences between radiologists and the deep learning algorithm, as well as between radiologists working in a fully blinded manner and those assisted by the deep learning algorithm. A 2-tailed *P* value<.05 was considered statistically significant. The statistical analysis of the above performance metrics was conducted using Python (version 3.8; Python Software Foundation).

## Results

### Construction of the Adrenal Masses Dataset

The construction of a dataset for Adrenal masses is exceedingly complex and entails a substantial workload. The adrenal masses dataset developed in this study consists of 6 categories, divided into two parts: (1) an internal dataset and (2) an external dataset. The internal dataset consists of 24,054 CT images from 343 patients with adrenal masses at the Second Affiliated Hospital of Kunming Medical University, while the external dataset comprises 12,857 CT images from 173 patients with adrenal masses at the First People’s Hospital of Yunnan Province. The inclusion and exclusion criteria of patients are illustrated in Figure S1 in Multimedia Appendix 1 [20,21], while the based characteristics of patients are outlined in Table 2. Then, we invited 3 radiologists with more than 20 years of experience to annotate all 36,911 CT images using the bounding box approach, an illustrative representation of the annotations for the 6 categories of adrenal tumors at different phases is provided in Figure S2 in Multimedia Appendix 1 [20,21]. Finally, we partitioned the internal dataset into training and validation sets in a ratio of 9:1, the breakdown by category is shown in Figure S3 in Multimedia Appendix 1 [20,21] and Table 3.

**Table 2:** Patient characteristics.

Dataset		AA^a^		PCC^b^		AM^c^		AC^d^		AGN^e^		ACC^f^	
		Internal	External	Internal	External	Internal	External	Internal	External	Internal	External	Internal	External
Patients, n		249	111	35	38	19	7	22	8	11	5	7	4
**Age (years)**													
	Mean (SD)	49.3 (10.8)	47.5 (11.3)	44.3 (14.9)	45.8 (16.5)	54.7 (8.2)	53.4 (12.7)	45.4 (13.1)	39.5 (15.4)	36.7 (15.8)	36.4 (17.2)	49.3 (12.4)	53.3 (6.1)
	Range	16-76	12-72	9-72	18-84	37-65	30-68	18-76	21-62	5-57	7-51	30-66	48-62
**Sex, n (%)**													
	Male	125 (50.2)	49 (44.1)	17 (48.6)	14 (36.8)	13 (68.4)	3 (42.9)	9 (40.9)	4 (50.0)	9 (81.8)	4 (80.0)	2 (28.6)	2 (50.0)
	Female	124 (49.8)	62 (55.9)	18 (51.4)	24 (63.2)	6 (31.6)	4 (57.1)	13 (59.1)	4 (50.0)	2 (18.2)	1 (20.0)	5 (71.4)	2 (50.0)

^a^AA: adrenocortical adenoma.

^b^PCC: pheochromocytoma.

^c^AM: adrenal myelolipoma.

^d^AC: adrenal cyst.

^e^AGN: adrenal ganglioneuroma.

^f^ACC: adrenocortical carcinoma.

**Table 3:** Summary of patient imaging dataset.

	Total number of images	Number of images in the internal training set	Number of images in the internal validation set	Number of images in the external test set
Total number of images	36,911	21,648	2406	12,857
AA^a^	15,839	9783	1087	4969
PCC^b^	9177	4541	505	4131
AM^c^	4563	2960	329	1274
AC^d^	3381	1931	215	1235
AGN^e^	1885	1109	123	653
ACC^f^	2066	1324	147	595

^a^AA: adrenocortical adenoma.

^b^PCC: pheochromocytoma.

^c^AM: adrenal myelolipoma.

^d^AC: adrenal cyst.

^e^AGN: adrenal ganglioneuroma.

^f^ACC: adrenocortical carcinoma.

### Performance of MA-YOLO Model

**Figure 3:**
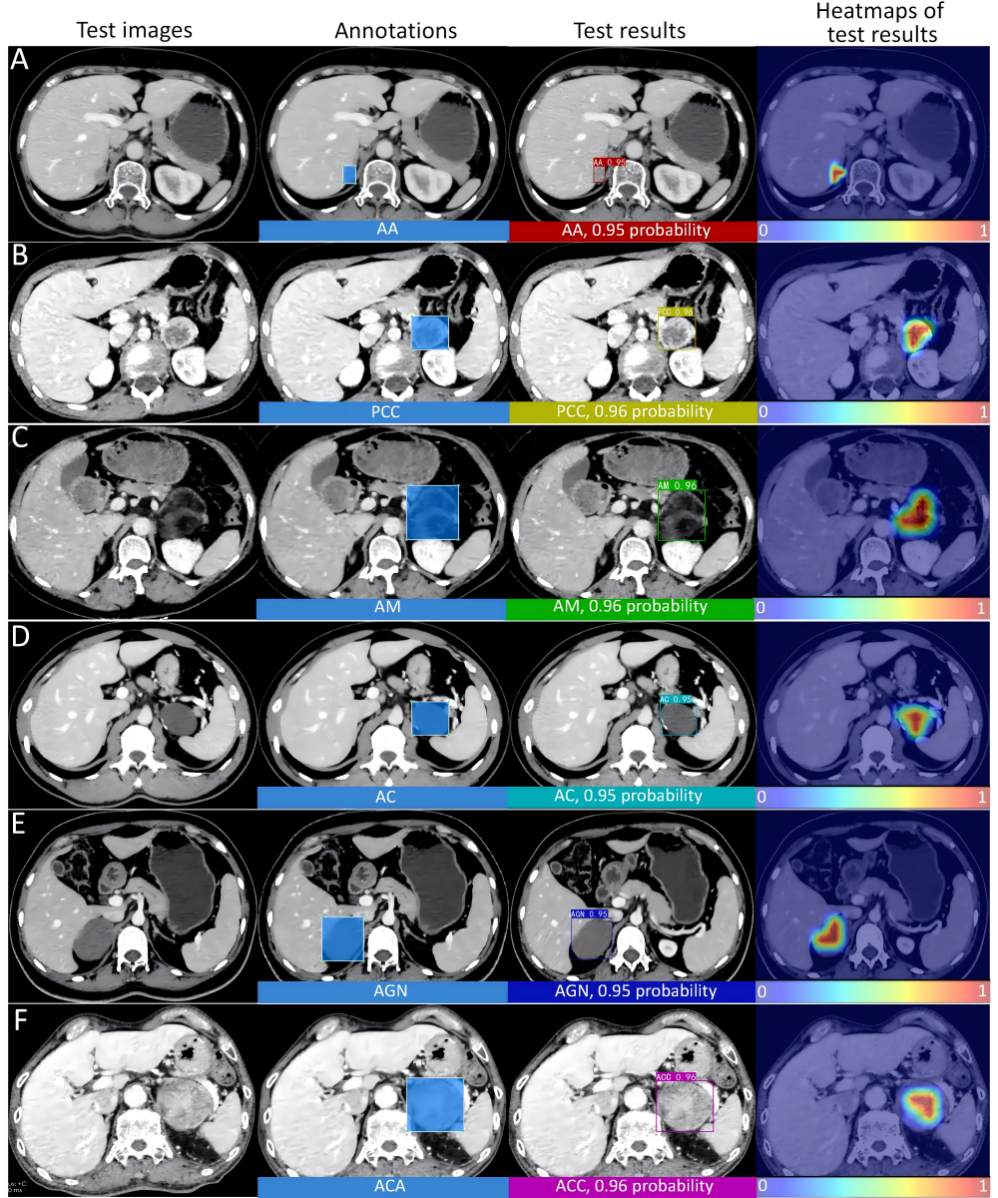
Original test images and images with annotations of 6 types of adrenal masses, test results based on the MA-YOLO model, and heat maps. (A) AA, (B) PCC, (C) AM, (D) AC, (E) AGN, and (F) ACC. AA: adrenocortical adenoma; AC: adrenal cyst; ACC: adrenocortical carcinoma; AGN: adrenal ganglioneuroma; AM: adrenal myelolipoma; MA-YOLO: Multi-Attention YOLO; PCC: pheochromocytoma.

Following training and validation on the internal dataset, the MA-YOLO model proposed in this study was evaluated on the external test set. Figure 3 shows the test results of 6 types of adrenal masses under the MA-YOLO model and the heat map of the model’s focus area.

**Figure 4:**
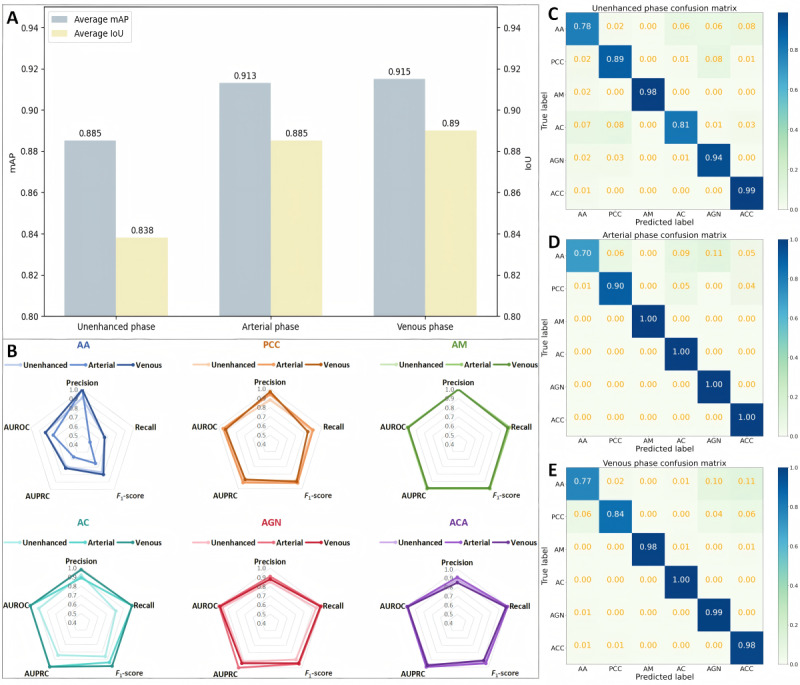
Performance of Multi-Attention YOLO (MA-YOLO) model on the external test set. (A) Average mAP and IoU for localizing and classifying the 6 types of adrenal masses across different phases. (B) Radar charts of classification performance for the 6 types of adrenal masses across 3 phases. Confusion matrices for classifying the 6 types of adrenal masses across (C) unenhanced, (D) arterial, and (E) venous phases. AA: adrenocortical adenoma; AC: adrenal cyst; ACC: adrenocortical carcinoma; AGN: adrenal ganglioneuroma; AM: adrenal myelolipoma; IoU: intersection over union; mAP: mean average precision; PCC: pheochromocytoma.

In order to evaluate the accuracy of the model in localizing adrenal masses, we used the IoU metric, and used metrics such as mAP, AUC, Recall, Confusion Matrix, and *F*_1_-score to assess the accuracy of the model in classifying the 6 types of adrenal masses. The results of the evaluation are shown in Figure 4.

Figure 4A illustrates the average mAP and IoU of the MA-YOLO model for localizing and classifying the 6 types of adrenal masses across different phases. Overall, the MA-YOLO model demonstrates satisfactory localization and classification performance across 3 phases. However, arterial and venous phases exhibit superior performance compared with the unenhanced phase. To provide a comprehensive presentation of the model testing results, in Figure 4B, we depict the classification performance of the MA-YOLO model for the 6 types of adrenal masses across 3 phases using radar charts. Figures 4C-Figures 4E presents the confusion matrices of the MA-YOLO model for classifying the 6 types of adrenal masses across different phases. It can be observed that the overall classification of the MA-YOLO model is effective in all 3 phases, except that the classification of AA is slightly inferior to that of the other categories.

The results indicate that the MA-YOLO model proposed in this study can accurately localize and classify the 6 types of adrenal masses, with relatively optimal performance observed in localization and classification by the venous phase model.

### Performance Comparison Between the MA-YOLO Model and Other Models

The MA-YOLO model proposed in this study is built upon the YOLO framework and incorporates various attention mechanisms. In order to validate the superior performance of the MA-YOLO model, this study adopts the YOLO V3, YOLO V5, YOLO V7, Fast R-CNN, and Efficientdet models as comparative models, which differ from each other in terms of the backbone network structure and optimization strategies. These models were trained using the same training set, hyperparameters, and subsequently tested on the same test set. The comparative experimental results are presented in Figures S4-S6 and Table S2 in Multimedia Appendix 1 [20,21].

For the overall assessment of model localization accuracy, we used the IoU metric. Among comparison models, the YOLO V3 model achieves IoU values of 0.717, 0.838, and 0.787 for the unenhanced phase, arterial phase, and venous phase respectively. The IoU values for the YOLO V5 model are 0.835, 0.859, and 0.847. The IoU values for the YOLO V7 model are 0.811, 0.865, and 0.873. The IoU values for the Fast R-CNN model are 0.550, 0.770, and 0.764, while for the Efficientdet model, they are 0.820, 0.840, and 0.870. The MA-YOLO model proposed in this study achieves IoU values of 0.838, 0.885, and 0.890 respectively, all of which are optimal. For the overall assessment of model classification accuracy, we used the mAP metric. Specifically, the YOLO V3 model achieves mA*P* values of 0.826, 0.903, and 0.875 for the unenhanced phase, arterial phase, and venous phase, respectively. Whereas, the mA*P* values of YOLO V5 model are 0.894, 0.889, and 0.883 and the mA*P* values of YOLO V7 model are 0.879, 0.908, and 0.905, respectively. In comparison, the MA-YOLO model proposed in this study achieves mA*P* values of 0.885, 0.913, and 0.915, demonstrating relatively superior performance.

The comparison experiments indicate that the MA-YOLO model proposed in this study can accurately localize and classify the 6 types of adrenal masses, and its performance generally surpasses that of other models.

### Performance Comparison Between the MA-YOLO Model and Physicians and Its Clinical Evaluation

**Figure 5:**
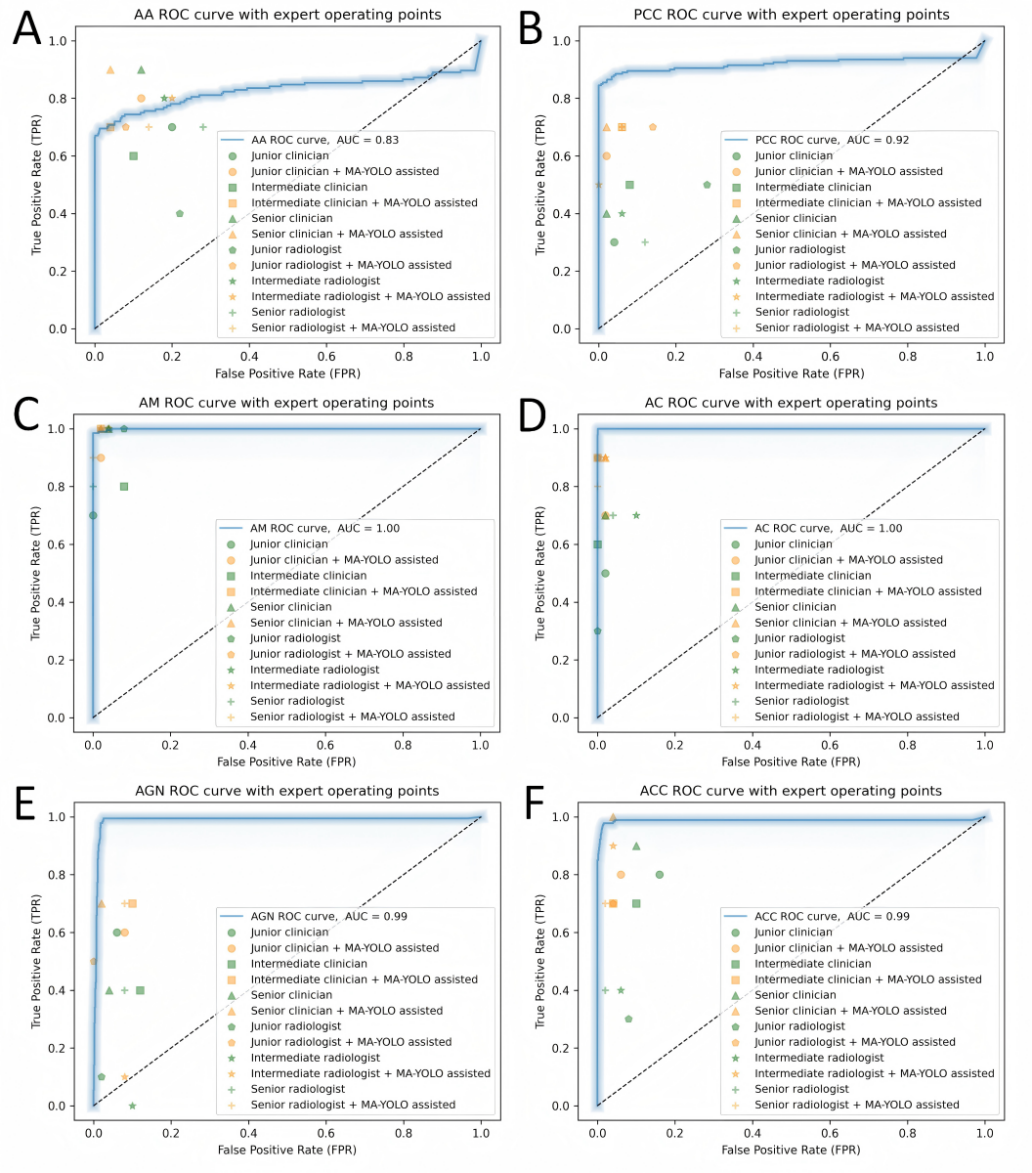
ROC curves for each category generated by the MA-YOLO model and expert operating points for physicians’ classification before and after model assistance. (A) AA, (B) PCC, (C) AM, (D) AC, (E) AGN, and (F) ACC. AA: adrenocortical adenoma; AC: adrenal cyst; ACC: adrenocortical carcinoma; AGN: adrenal ganglioneuroma; AM: adrenal myelolipoma; AUC: area under the receiver operating characteristic curve; MA-YOLO: Multi-Attention YOLO; PCC: pheochromocytoma; ROC: receiver operating characteristic.

The ROC curves for the classification diagnosis of 6 types of adrenal masses using the MA-YOLO model are shown in Figure 5. On these ROC curves, we marked the expert operating points of 6 physicians before and after assistance from the MA-YOLO model. To evaluate the superiority of the MA-YOLO model and physicians’ independent diagnoses, as well as the significance of the model’s improvement in physicians’ diagnostic performance, this study used the DeLong test [23,24]. The comparison between the MA-YOLO model and physicians’ independent diagnoses is presented in Multimedia Appendix 2, while the comparison between physicians’ independent diagnoses and their diagnoses with model assistance is shown in Multimedia Appendix 3. Table 4 lists the comparison of physicians’ diagnosis times before and after model assistance, as well as the diagnosis time of the model. The DeLong test results indicated that, except for the AM category, significant differences were observed in all categories across one or more comparison groups between the model and physicians’ independent diagnoses. Excluding the senior physician group, the MA-YOLO model demonstrated significantly higher diagnostic efficiency for PCC and ACC categories in the other 5 comparison groups. Additionally, its diagnostic efficiency for AC and AGN categories was significantly better than physicians’ independent diagnoses in at least half of the comparison groups, highlighting the model’s excellent diagnostic performance for these 4 types of adrenal masses. Even in the AA category, where the model’s diagnostic performance was the lowest, 2 comparison groups showed significant differences. Thus, it can be concluded that, except for the AM category, the MA-YOLO model demonstrated superior diagnostic efficiency over physicians for the other 5 types of adrenal masses.

Moreover, the DeLong test results showed that, compared with physicians’ independent diagnoses, the diagnostic efficiency of the other 4 mass categories significantly improved in 3 out of 6 comparison groups under the assistance of the MA-YOLO model, excluding AM and AGN categories. For the AGN category, significant improvements were observed in 2 of the 6 comparison groups. Furthermore, as shown in Figure 5, under optimal model assistance, nearly all physicians maintained or improved their accuracy in classifying adrenal masses across all categories, achieving significant improvements in the classification diagnosis of the aforementioned mass categories.

Finally, the decision curve reflecting the clinical net benefit of the MA-YOLO model is shown in Figure S7 in Multimedia Appendix 1 [20,21].

**Table 4:** Average diagnostic duration for urologists and radiologists for the classification of 6 types of adrenal masses.

Diagnostician	Without assistance (average diagnostic time per patient in seconds)	With MA-YOLO^a^ assistance (average diagnostic time per patient in seconds)	Multi-Attention YOLO (average diagnostic time per patient in seconds)
Junior radiologist	10.97	7.93	—^b^
Intermediate radiologist	12.73	11.65	—
Senior radiologist	16.45	17.98	—
Junior clinician	13.85	12.58	—
Intermediate clinician	17.54	15.00	—
Senior clinician	13.80	11.52	—
AI^c^	—	—	0.05

^a^MA-YOLO: Multi-Attention YOLO.

^b^Not applicable.

^c^AI: artificial intelligence.

## Discussion

### Principal Findings

The adrenal glands are situated in a unique anatomical position, characterized by their small size and proximity to various neighboring organs, making the interpretation of abdominal CT scan images complex. Consequently, physicians diagnosing adrenal masses need to make comprehensive judgments by comparing images from different phases and overall views. However, the results of classifying these masses are often unsatisfactory, and due to the influence of these factors, machine learning also encounters challenges in handling such complex images. This multicenter study demonstrates that the MA-YOLO–based deep learning algorithm can more accurately identify adrenal masses using contrast-enhanced abdominal CT scans, thereby improving diagnostic efficiency for clinicians with artificial intelligence (AI) assistance and holding the potential to transform current clinical practice.

### Comparison to Prior Work

Previous research related to adrenal machine learning has predominantly focused on tasks such as the automatic segmentation of adrenal glands, discrimination between normal adrenal glands and those containing masses, and classification of certain types of adrenal masses [16-18,25,26]. However, most of these studies are single-center investigations and seldom classify based on accurate pathological results. This limitation hinders the demonstration of the model’s generalizability and fails to provide convincing classification criteria for readers. In addition, as mentioned above, the MA-YOLO model proposed in this study constructs Focus module, ConvAttention module, Transition module, and Convolutional Block Attention module on the basis of the YOLO model, which enables the model to extract and use the key information in the image more efficiently, and is able to better fusion of local and global information in the image, and at the same time can reduce the computational complexity, improve the training and inference efficiency of the model, and has better generalization. In contrast, the aforementioned studies generally adopt U-Net, DensNet, Convolution, or a combination of both models, and although they all achieve better results in their respective tasks, they all lack the attention mechanism with adaptive ability, and have certain limitations in feature extraction, computational efficiency, and model generalization.

This study is the first deep learning model for the automatic detection and classification of adrenal masses based on multicenter data. The application of external data confirmed the generalizability of the MA-YOLO model. To evaluate the performance of the MA-YOLO model, we conducted comparative experiments, selecting YOLO V3, V5, and V7 models representing different stages of YOLO development, as well as the widely recognized Faster R-CNN and EfficientDet object detection algorithms. The comparative experimental results with the aforementioned 5 models indicate that the MA-YOLO model exhibits superior overall performance (Table S1 in Multimedia Appendix 1 [20,21]). This can likely be attributed to the integration of multiple spatial and channel attention modules in the MA-YOLO model, enabling it to better capture critical information in both spatial and channel dimensions. Notably, the improvement is more pronounced for certain specific tumors, such as AA and PCC. Both AA and PCC account for the vast majority of adrenal masses (>90%) and, with the exception of nonfunctional AA, typically require surgical intervention. Therefore, the aforementioned improvements are of significant importance for AI diagnostic models of adrenal masses. From another perspective, it can assist clinicians in quickly locating masses, saving time in image interpretation, and also help patients understand their condition, thereby alleviating anxiety.

### Practical Value of the MA-YOLO Model

The MA-YOLO model demonstrates diagnostic accuracy for classifying 6 tumor types that are greater than or equal to all 6 physicians. Its practical value is primarily reflected in the significant improvement in diagnostic accuracy for certain specific tumor categories compared with the 6 physicians (Multimedia Appendix 2). The improvement is particularly significant in the 4 tumor types: PCC, ACC, AC, and AGN. Certain characteristics of these tumors in actual diagnostic practice, such as similar imaging features among them, the relative rarity of ACC and AGN leading to insufficient clinical experience, and other factors [27,28], contribute to reduced diagnostic accuracy by physicians when assessing these tumor categories in real-world scenarios. Deep learning algorithms can effectively address the aforementioned issues, thereby improving diagnostic accuracy. In clinical practice, especially when dealing with the 4 tumor types mentioned, accurate preoperative diagnosis is essential. For example, asymptomatic or small ACs may be monitored through observation rather than undergoing unnecessary surgical intervention under uncertain conditions [29]. PCC and AGN require surgical excision, and adequate preoperative preparation is essential to prevent significant hemodynamic fluctuations during surgery [30]. Patients suspected of having ACC need further systemic evaluation and may require a unilateral adrenalectomy distinct from other tumor categories, potentially accompanied by lymph node dissection. Additionally, postoperative adjuvant therapy may be necessary [31]. On the other hand, due to the current level of trust that physicians place in AI results, along with ethical and safety concerns, it is unrealistic to fully delegate decision-making to AI. Therefore, we use AI results as an adjunct to the clinician’s judgment. The results indicate that with AI assistance, physicians show a universally significant improvement in diagnostic accuracy for 4 tumor types: AA, PCC, AC, and ACC (Multimedia Appendix 3). This holds substantial practical significance and provides a realistic reference for future clinical applications.

### Future Directions

It is worth noting that in terms of recognition and classification, the model’s performance in AA is slightly inferior to that of the other 5 tumor types. This may be due to the smaller average diameter of AA compared with the other tumor categories. Some studies have found that deep learning may lack sufficient feature extraction capabilities to identify small lesions. For instance, Bi et al [25] discovered that in the CT cross-sectional images of certain adrenal lesions, the first or last layer often displays smaller tumor diameters, which may lack sufficient lesion information for feature extraction, leading to a decrease in recognition and classification accuracy for these images. Additionally, while examining the AA test set images, we observed that some smaller-diameter AAs were not successfully identified. Therefore, we randomly selected 200 images of AAs with diameters greater than 2 cm for further testing and found a significant improvement in localization efficiency, with an IoU of 0.806, compared with an IoU of 0.779 for the AA images in the test set without diameter screening. Furthermore, the improvement in localization efficiency also impacted classification accuracy, as tumors that were not successfully localized in the images could not be classified for prediction. Additionally, considering that AA includes both fat-rich and fat-poor adenomas, we selected 150 images each of fat-rich (average CT value of the largest tumor diameter <10 HU) and fat-poor (average CT value of the largest tumor diameter >10 HU) adenomas for further testing, using the internationally recognized CT value threshold of 10 HU [32-35]. The MA-YOLO model achieved an AUC of 0.87 in diagnosing fat-poor adenomas and 0.80 in diagnosing fat-rich adenomas. Considering low-density lesions such as AC and AGN, the low density in the images implies lower pixel density, which can affect the model’s localization and classification results. Therefore, we believe that the slight decline in the model’s accuracy in AA classification and diagnosis may be related to their smaller diameter and heterogeneous density.

Although the results in the unenhanced phase of this study were slightly lower than those in the other 2 phases, both the mAP and IoU still exceeded 0.8. Considering the following three factors—(1) the economic burden of contrast-enhanced CT scans on patients, (2) the potential effects of contrast agent injection, and (3) the significantly longer examination time required for contrast-enhanced CT compared with unenhanced CT—future research should focus more on improving the performance of deep learning algorithms in unenhanced CT scans. Based on existing guidelines, AI assistance significantly reduces the extent and complexity of patient examinations while ensuring diagnostic accuracy, which holds significant value for both clinicians and patients. Additionally, as the detection rate of AI (artificial intelligence) continues to improve annually, it suggests the potential need for large-scale adrenal screening in the future. Mature AI algorithms can assist clinicians in preliminary localization and classification tasks, significantly reducing their workload and improving efficiency.

### Study Limitations

This study still has several limitations. First, it is a retrospective study, and future research should collect prospective data to further validate the performance of the MA-YOLO model. Second, this study was based on 2D images for training and did not incorporate 3D modeling of all patients’ tumors or conduct multimodal analysis with clinical data. Multimodal and multidimensional analysis is the future direction of medical AI, and further research in these areas is necessary. Third, we only included adrenal masses with a diameter greater than 1 cm. Although this is the accepted definition for incidental adrenal nodules, some adrenal masses may be smaller than 1 cm. Future research may need to further explore the localization and classification of adrenal nodules smaller than 1 cm. Finally, this study only examined the performance of the Multi-Attention YOLO algorithm in the localization and classification of adrenal masses and did not include imaging of patients with tumors involving adjacent organs of the adrenal gland. The adrenal gland is adjacent to several organs, and tumors, such as those of the liver or upper pole of the kidney, are commonly found around the adrenal gland. These masses may interfere with the automatic localization and classification of adrenal masses. Therefore, future studies should include imaging of patients with tumors adjacent to the adrenal gland to further explore the performance of the MA-YOLO model in complex scenarios.

### Conclusions

In conclusion, this study collected multicenter abdominal multiphase CT data and established a dataset of adrenal masses. We proposed the MA-YOLO model based on the multiattention mechanism and the YOLO framework, which can quickly and accurately automatically localize and classify 6 common types of adrenal masses. With the assistance of the model, the diagnostic performance of physicians can be effectively improved, providing meaningful preoperative reference for physicians.

## Data Availability

The datasets generated or analyzed during this study are available from the corresponding author on reasonable request.

## Appendix

Multimedia Appendix 1 Additional methods, figures, and tables.

Multimedia Appendix 2 Comparison of performance for each subclass of adrenal masses between independent diagnosis by physicians and by the model.

Multimedia Appendix 3 Comparison of diagnostic performance for each subclass of adrenal masses between independent diagnoses by physicians or with assistance from the Multi-Attention YOLO (MA-YOLO) model.

## Abbreviations

AA: adrenocortical adenoma
AC: adrenal cyst
ACC: adrenocortical carcinoma
AGN: adrenal ganglioneuroma
AI: artificial intelligence
AM: adrenal myelolipoma
AUC: area under the receiver operating characteristic curve
CT: computed tomography
HU: Hounsfield units
IoU: intersection over union
mAP: mean average precision
MA-YOLO: Multi-Attention YOLO
PCC: pheochromocytoma
R-CNN: region-based convolutional neural network
ROC: receiver operating characteristic
