# Supplementary material for: Localization and Classification of Adrenal Masses in Multiphase Computed Tomography: Retrospective Study
Source: J Med Internet Res. 2025 Apr 24;27:e65937. doi: 10.2196/65937 (PMC12062765; doi:10.2196/65937)
Supplement: Multimedia Appendix 1 [file jmir_v27i1e65937_app1.docx]

**Supplementary**

Localization and classification of adrenal masses in multi-phase CT: A deep learning framework

Liuyang Yang^1,2,3*^, Xinzhang Zhang^1,4,5,6^**^*^**, Zhenhui Li^1*^, Jian Wang^2*^, Yiwen Zhang^4,6^, Liyu Shan^1^, Xin Shi^6,7^, Yapeng Si^4,5,6^, Shuailong Wang^4,5,6^, Lin Li^4,5,6^, Ping Wu^4,5,6^, Ning Xu^1^, Lizhu Liu^1^, Junfeng Yang^4,5^, Jinjun Leng^4,5^, Maolin Yang^4,5^, Zhuorui Zhang^4,5^, Junfeng Wang^4^, Xingxiang Dong^1^, Guangjun Yang^1^, Ruiying Yan^1^, Wei Li^8^, Zhimin Liu^1^, Wenliang Li^1^

^1^Yunnan Cancer Hospital, The Third Affiliated Hospital of Kunming Medical University, Kunming, Yunnan, China

^2^School of Data Science, Fudan University, Shanghai, China.

^3^School of Population Medicine and Public Health, Chinese Academy of Medical Sciences & Peking Union Medical College, Beijing, China

^4^The Affiliated Hospital of Kunming University of Science and Technology, Kunming, Yunnan, China

^5^Department of Urology, The First People's Hospital of Yunnan Province, Kunming, Yunnan, China

^6^Medical School, Kunming University of Science and Technology, Kunming, Yunnan, China

^7^Department of Urology, Second Affiliated Hospital of Kunming Medical University, Kunming, Yunnan

^8^Kunming Medical University, Kunming, Yunnan, China

^*^These authors contributed equality.

**Corresponding Author:**

Wenliang Li, M.D.‌‌

Yunnan Cancer Hospital, The Third Affiliated Hospital of Kunming Medical University

No. 519, Kunzhou Road, Xishan District

Kunming, Yunnan, 650118

China

Phone: +86087168189037

Email: [liwenliang@kmmu.edu.cn](mailto:liwenliang@kmmu.edu.cn)

**Supplementary Material**

**CONTENTS**

**1. Methods**

**1.1 Development of the MA-YOLO model**

**1.2 Data augmentation techniques**

**1.3 Training strategy and hyperparameter selection**

**2. Supplementary figures**

**2.1 Figure S1**

**2.2 Figure S2**

**2.3 Figure S3**

**2.4 Figure S4**

**2.5 Figure S5**

**2.6 Figure S6**

**2.7 Figure S7**

**3. Supplementary tables**

**3.1 Table S1**

**3.2 Table S2**

**4. References**

**1. Methods**

1.1 Development of the MA-YOLO model

As shown in Figure. 2, in this study, Focus Module, ConvAttention Module, Transition Module and Convolutional Block Attention Module (CBAM), were used to construct the Multi-attention. The specific operation of Focus Module is to take a value every other pixel in the 2D grayscale image, at this time, multiple independent feature layers are obtained, and then multiple independent feature layers are spliced on the channel to expand the number of input channels, at this time, the width and height information of the 2D grayscale image is distributed into multi-dimensional channels, which is convenient for the subsequent use of multi-attention mechanisms. ConvAttention Module is based on Convolution 2D and adds Squeeze and Excitation Attention Module[1], which is used to take into account the spatial and channel information, focus on extracting the key channel information while extracting the spatial information and suppressing the information of non-key channels. The Transition Module consists of multiple ConvAttention Modules, one of which adopts multi-layer ConvAttention Modules for deep extraction of data features to improve the degree of model feature extraction by increasing the depth of the modules, and the other part only performs residual connection after simple feature extraction, which can effectively alleviate the problem of spatial and channel information extraction in deep neural networks due to the lack of spatial and spatial information. CBAM Module consists of Channel Attention and Spatial Attention[2], Channel Attention is used to adaptively adjust the feature responses of different channels to improve the sensitivity to different features. Spatial Attention is used to adaptively adjust the feature responses of different spatial locations to improve the sensitivity to the target location.

In the Focus module of this study, a (3x3) convolution kernel with a stride of 1 (Stride = 1) is used for the Convolution2D operation. Specifically, this operation samples every other pixel from the 2D grayscale image, resulting in multiple independent feature layers. These feature layers are then concatenated along the channels, increasing the number of input channels. As a result, the width and height information of the 2D grayscale image is distributed across multiple channels, which facilitates the model in finely extracting local features and enhances its ability to detect small objects. At the same time, it preserves resolution, preventing the loss of important details. Additionally, this approach reduces computational complexity, improving the overall efficiency of the model, and further enhances feature representation through spatial information transformation optimization.

In the ConvAttention Module of this study, Convolution2D uses a (3x3) convolution kernel with a stride of 1. This operation enables efficient dimensionality reduction while fusing channel information, mitigating overfitting, and preserving resolution. It enhances the model's ability to understand complex targets and improves detection of small lesions, effectively increasing the model's recall rate and accuracy. The attention mechanism’s Global Pooling, with an output size of (1x1), helps the network focus on key areas within the image, improving its sensitivity to small lesions and other detailed targets. This also boosts the model's generalization ability and enhances its adaptability to cross-sample or cross-device differences.

In the Transition Module of this study, ConvAttention Module 1 and ConvAttention Module 3 use (3x3) and (1x1) convolution kernels, respectively. The (3x3) convolution kernel extracts local texture features, enhancing the boundary information of targets while expanding the receptive field to improve detection performance for medium and small targets. The (1x1) convolution kernel reduces redundant features, lowers computational complexity, and improves feature representation across channels, making the model adaptable to diverse targets. The collaboration of both kernels effectively enhances the recognition of small lesions, boundary localization, and background suppression.

In the Channel Attention Module of this study, the Shared Dense Scaling ratio is set to 16, allowing dynamic adjustment of channel weights to highlight key channels while suppressing redundant ones. This effectively improves the detection of small lesions in complex backgrounds. In the Spatial Attention Module, a (6x6) convolution kernel is used, which captures a broader range of spatial features, enabling the model to focus on prominent areas in the image and effectively attend to larger lesions. The combination of both modules allows the model to simultaneously focus on both global and local features, thereby enhancing its ability to detect lesions of varying sizes.

To ensure that the size of the input model images remains consistent, all the images are firstly resized to 640x640 pixels, and then, input to the Focus Module which distributes the pixel information from the single-channel two-dimensional grayscale image plane to the three-dimensional multi-dimensional channels, and then, through one ConvAttention Module and four ConvAttention Module+Transition Module+CABM Module to further extract image features in spatial and channel dimensions, respectively. Then, the features extracted by the backbone network and the features extracted by ConvAttention Module and ConvAttention Module+Transition Module are spliced and fused, so that the fused results have multi-scale features. Finally, the output results are obtained after the fusion of the Transition Module and the splicing of the Transition Module features. More detailed parameter settings can be found in Table S1.

Before model training, we randomly split the training set, the validation set in the ratio of 9:1 to the dataset. When the model is trained, we set the batch size to 8, the number of training epochs to 150, choose the stochastic gradient descent (SGD) strategy as the optimizer, the initial learning rate is set to 0.01, and Cosine annealing method is used for learning rate decay. Mosaic Data Augmentation method is also used to enable the model to better recognize small targets. All the training, validation and testing processes are performed on one NVIDIA RTX 4090 Graphics Processing Unit (GPU) with 24G memory.

1.2 Data augmentation techniques

In this study, to enhance the generalization ability of the MA-YOLO model in the detection and classification of adrenal masses, we employed the following specific data augmentation techniques, which were carefully selected and optimized based on the characteristics of the task:

(1)Mosaic data augmentation

Mosaic data augmentation is a technique that combines multiple images into a single new image. This method randomly combines different images and their corresponding labels during training, altering the distribution and proportions of the objects in the images to simulate complex scenarios in diverse settings. It can expand the diversity of object scales and background distributions, providing a wider range of object arrangements, which prevents overfitting and effectively improves the model's robustness and generalization ability.

For the adrenal tumor detection task focused on in this study, the target regions exhibit considerable diversity in morphology and distribution. Mosaic data augmentation can help the model better adapt to variations in tumor shapes, positions, and sizes, while enhancing robustness against complex backgrounds.

(2)Mixup data augmentation

Mixup data augmentation involves linearly combining two images and adjusting their labels accordingly to generate synthetic training samples. This technique introduces a linear combination of samples, increasing data diversity, smoothing label distributions, and reducing the risk of overfitting. It enhances the model's ability to discriminate against minority and boundary samples, improving the model's adaptability to boundary and noisy samples.

(3)Other fundamental data augmentation techniques.

In addition to the aforementioned specific augmentation methods, we also employed other basic augmentation techniques, such as random rotation and flipping, random cropping and scaling, and Gaussian noise addition. These methods further enhance the model's generalization capability.

1.3 Training strategy and hyperparameter selection

In this study, to ensure the efficiency of the training process and optimize model performance, we comprehensively considered hardware capabilities, dataset size, and model complexity, and made the following settings and optimizations for batch size, training epochs, and learning rate.

(1) The batch size was set to 8.

In this study, since the model was trained on an NVIDIA RTX 4090 24GB GPU with limited memory, we selected a batch size of 8 after considering the GPU memory usage, model parameter size, and input image resolution. This batch size allowed us to efficiently utilize GPU memory while avoiding memory overflow that could interrupt training, striking a good balance between training efficiency and performance optimization. Additionally, the smaller batch size helped improve the stability of the convergence process, particularly in this study where the dataset was large and class imbalance existed.

(2) The training epochs were initially set to 150, with the implementation of an Early Stopping strategy to optimize the training process.

Based on common experience in object detection tasks, the training epoch was set to 150, which typically ensures that the model learns features sufficiently from the dataset while avoiding overfitting. Additionally, to prevent prolonged training times and potential overfitting, we implemented an Early Stopping strategy. This strategy halts the training process automatically if the performance on the validation set does not improve over 20 consecutive epochs. This approach ensures both efficient model training and stable performance. Through observations of training curves in small-scale experiments, it was found that 150 epochs was sufficient for the model to reach good convergence, while Early Stopping further minimized unnecessary training.

(3) The initial learning rate is set to 0.01, and the learning rate is optimized using the Cosine Annealing Learning Rate Schedule.

Based on previous model training experience, an initial learning rate of 0.01 is considered appropriate. This value ensures that the model learns initial features quickly while avoiding oscillations caused by an overly large learning rate. We verified this through the Learning Rate Range Test, which demonstrated that an initial learning rate of 0.01 allowed the loss function to decrease rapidly and stabilize. Additionally, we employed the Cosine Annealing Learning Rate Schedule to optimize the learning rate. This approach prevents the model from converging to a local optimum during the later stages of training by gradually decreasing the learning rate, helping to avoid premature convergence and improving the model's generalization ability.

**2. Supplementary figures**

2.1 Figure S1


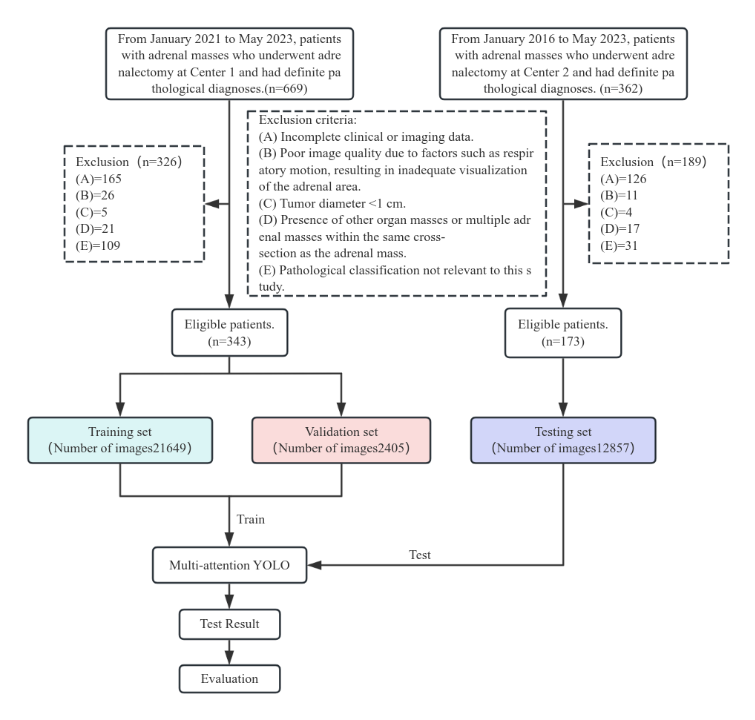


Figure S1. Patient inclusion and exclusion criteria and process

2.2 Figure S2


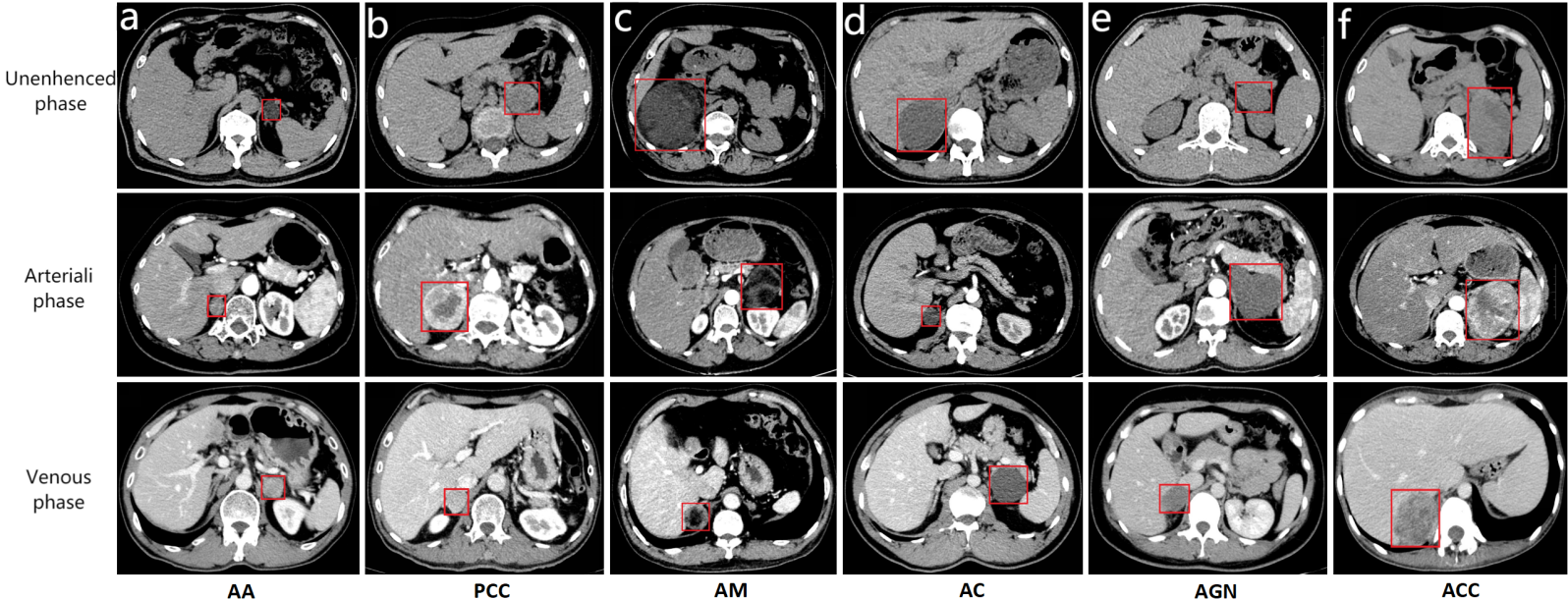
 Figure S2. Annotation of six types of adrenal masses across different phases. a AA, b PCC, c AM, d AC, e AGN, f ACC. Note: This figure presents the results of manual annotation, with one representative image selected for each category in each phase.

2.3 Figure S3


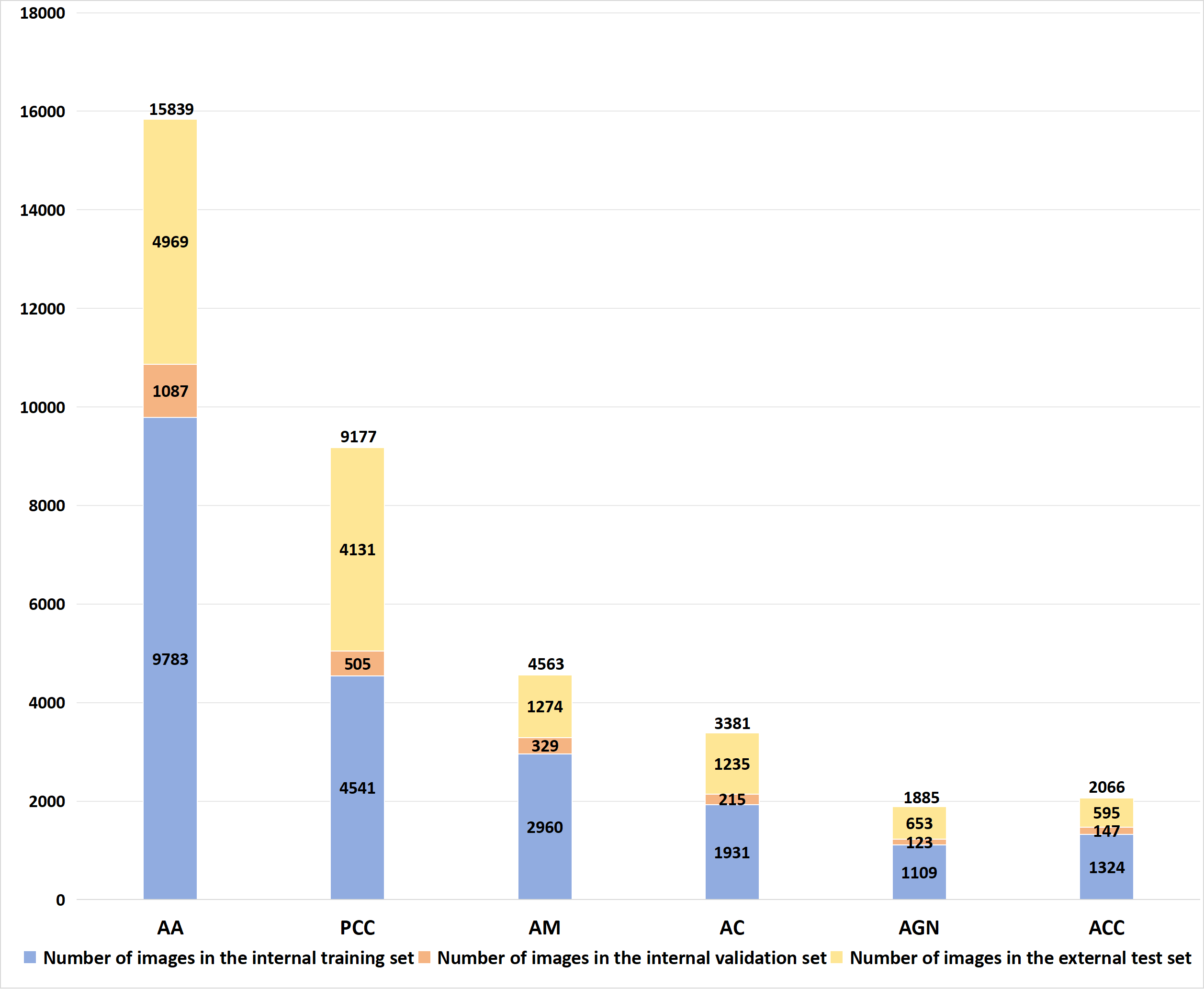


Figure S3. Stacked bar graph of the number of images in the internal training set, internal validation set, and external test set for the six classes of adrenal masses

2.4 Figure S4

**
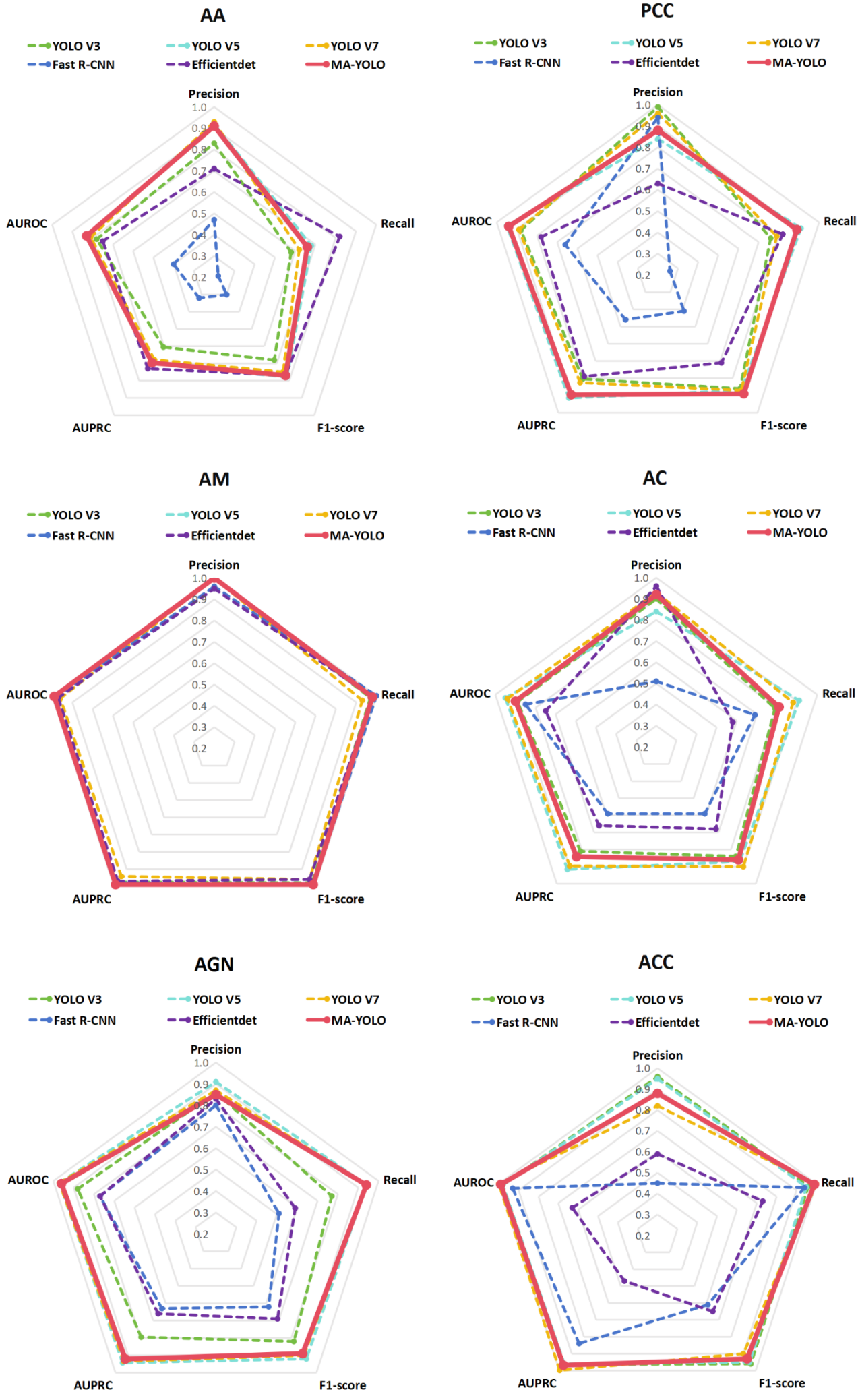
**

Figure S4. The classification performance of six models in the unenhanced phase.

2.5 Figure S5


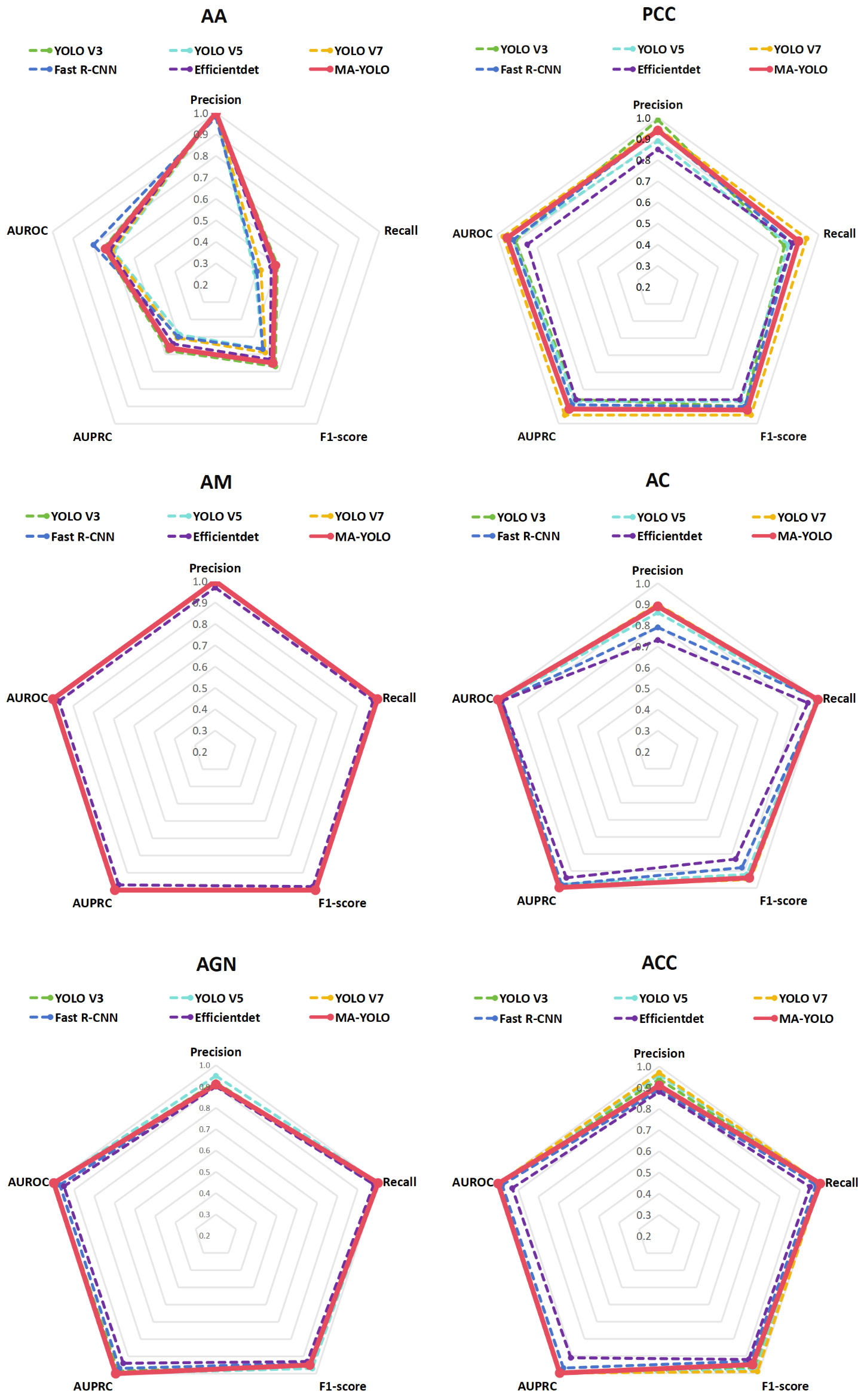


Figure S5. The classification performance of six models in the arterial phase.

2.6 Figure S6


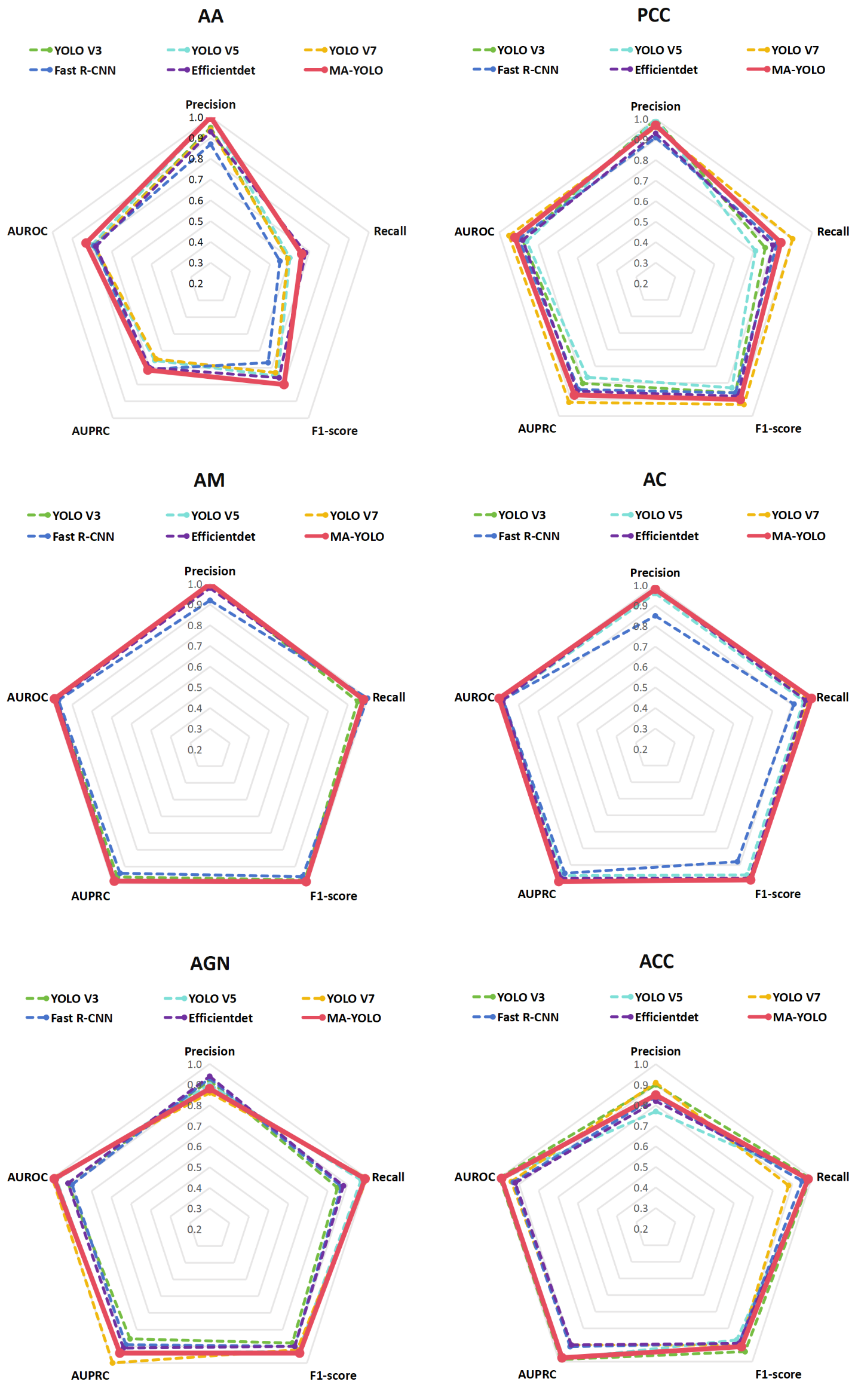


Figure S6. The classification performance of six models in the venous phase.

2.7 Figure S7


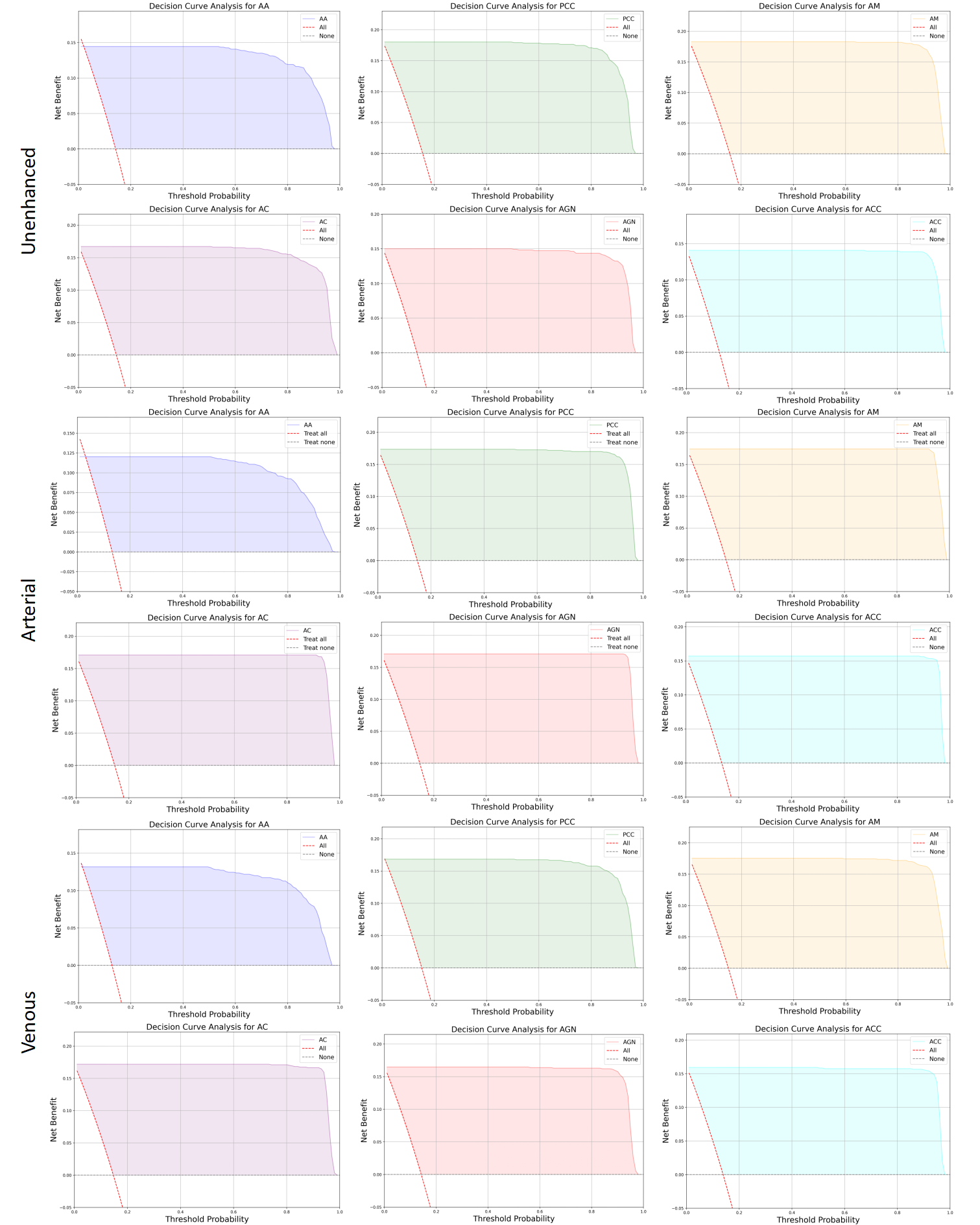


Figure S7. Clinical decision curves of the MA-YOLO model for diagnosing six tumor types in the unenhanced, arterial, and venous phases.

**3. Supplementary tables**

3.1 Table S1

Table S1 Detailed breakdown of the layers and parameters used in the MA-YOLO model

| Module name | Layer name | Parameter |
| --- | --- | --- |
| Focus Module | Convolution2D | Kernel = (3x3), Stride = 1 |
| ConvAttention Module | Convolution2D | Kernel = (3x3), Stride = 1 |
|  | Global Pooling | Output size = (1x1) |
| ConvAttention Module 1(Transition) | Convolution2D | Kernel = (1x1), Stride = 1 |
|  | Global Pooling | Output size = (1x1) |
| ConvAttention Module 3(Transition) | Convolution2D | Kernel = (3x3), Stride = 1 |
|  | Global Pooling | Output size = (1x1) |
| Channel Attention Module(CBAM) | Global Pooling | Output size = (1x1) |
|  | Average Pooling | Output size = (1x1) |
|  | Shared Dense | Scaling ratio = 16 |
| Spatial Attention Module(CBAM) | Global Pooling | Output size = (1x1) |
|  | Average Pooling | Output size = (1x1) |
|  | Convolution2D | Kernel = (6x6), Stride = 1 |

3.2 Table S2

Table S2. Comparison of the performance of MA-YOLO model with other models across different phases.

| Model | Phase | Classification | Precision | Recall | F1-score | AUPRC | AUROC | mAP | IoU |
| --- | --- | --- | --- | --- | --- | --- | --- | --- | --- |
| Yolo v3 | Unenhanced | AA | 0.83 | 0.58 | 0.68 | 0.605 | 0.78 | 0.826 | 0.717 |
|  |  | PCC | 0.99 | 0.76 | 0.86 | 0.804 | 0.88 |  |  |
|  |  | AM | 1.00 | 0.97 | 0.98 | 0.975 | 0.98 |  |  |
|  |  | AC | 0.90 | 0.79 | 0.84 | 0.810 | 0.89 |  |  |
|  |  | AGN | 0.86 | 0.77 | 0.82 | 0.795 | 0.88 |  |  |
|  |  | ACC | 0.96 | 0.96 | 0.96 | 0.965 | 0.98 |  |  |
|  | Arterial | AA | 1.00 | 0.50 | 0.67 | 0.579 | 0.75 | 0.903 | 0.838 |
|  |  | PCC | 0.99 | 0.83 | 0.90 | 0.859 | 0.91 |  |  |
|  |  | AM | 1.00 | 1.00 | 1.00 | 1.000 | 1.00 |  |  |
|  |  | AC | 0.89 | 1.00 | 0.94 | 0.997 | 1.00 |  |  |
|  |  | AGN | 0.92 | 0.99 | 0.96 | 0.984 | 1.00 |  |  |
|  |  | ACC | 0.94 | 1.00 | 0.97 | 1.000 | 1.00 |  |  |
|  | Venous | AA | 0.95 | 0.59 | 0.73 | 0.651 | 0.79 | 0.875 | 0.787 |
|  |  | PCC | 1.00 | 0.76 | 0.86 | 0.803 | 0.88 |  |  |
|  |  | AM | 1.00 | 0.95 | 0.98 | 0.962 | 0.98 |  |  |
|  |  | AC | 0.98 | 0.99 | 0.99 | 0.995 | 1.00 |  |  |
|  |  | AGN | 0.92 | 0.85 | 0.88 | 0.855 | 0.92 |  |  |
|  |  | ACC | 0.90 | 0.99 | 0.94 | 0.987 | 1.00 |  |  |
| Yolo v5 | Unenhanced | AA | 0.92 | 0.68 | 0.78 | 0.698 | 0.83 | 0.894 | 0.835 |
|  |  | PCC | 0.97 | 0.84 | 0.90 | 0.868 | 0.92 |  |  |
|  |  | AM | 1.00 | 0.98 | 0.99 | 0.984 | 0.99 |  |  |
|  |  | AC | 0.84 | 0.91 | 0.87 | 0.915 | 0.95 |  |  |
|  |  | AGN | 0.91 | 0.94 | 0.92 | 0.943 | 0.97 |  |  |
|  |  | ACC | 0.95 | 0.95 | 0.95 | 0.960 | 0.98 |  |  |
|  | Arterial | AA | 1.00 | 0.39 | 0.57 | 0.487 | 0.70 | 0.889 | 0.859 |
|  |  | PCC | 0.89 | 0.85 | 0.87 | 0.869 | 0.92 |  |  |
|  |  | AM | 1.00 | 1.00 | 1.00 | 1.000 | 1.00 |  |  |
|  |  | AC | 0.86 | 0.99 | 0.92 | 0.978 | 0.99 |  |  |
|  |  | AGN | 0.95 | 1.00 | 0.97 | 0.999 | 1.00 |  |  |
|  |  | ACC | 0.96 | 1.00 | 0.98 | 1.000 | 1.00 |  |  |
|  | Venous | AA | 1.00 | 0.60 | 0.75 | 0.657 | 0.80 | 0.883 | 0.847 |
|  |  | PCC | 1.00 | 0.71 | 0.83 | 0.766 | 0.86 |  |  |
|  |  | AM | 0.99 | 0.98 | 0.99 | 0.983 | 0.99 |  |  |
|  |  | AC | 0.96 | 0.96 | 0.96 | 0.965 | 0.98 |  |  |
|  |  | AGN | 0.90 | 0.97 | 0.93 | 0.943 | 0.98 |  |  |
|  |  | ACC | 0.77 | 0.99 | 0.87 | 0.983 | 0.99 |  |  |
| Yolo v7 | Unenhanced | AA | 0.93 | 0.62 | 0.75 | 0.678 | 0.81 | 0.879 | 0.811 |
|  |  | PCC | 0.96 | 0.79 | 0.87 | 0.825 | 0.89 |  |  |
|  |  | AM | 1.00 | 0.93 | 0.96 | 0.942 | 0.96 |  |  |
|  |  | AC | 0.93 | 0.88 | 0.90 | 0.896 | 0.94 |  |  |
|  |  | AGN | 0.87 | 0.94 | 0.90 | 0.934 | 0.97 |  |  |
|  |  | ACC | 0.82 | 1.00 | 0.90 | 0.998 | 1.00 |  |  |
|  | Arterial | AA | 1.00 | 0.42 | 0.59 | 0.506 | 0.71 | 0.908 | 0.865 |
|  |  | PCC | 0.95 | 0.94 | 0.95 | 0.950 | 0.97 |  |  |
|  |  | AM | 1.00 | 1.00 | 1.00 | 1.000 | 1.00 |  |  |
|  |  | AC | 0.90 | 1.00 | 0.95 | 0.992 | 1.00 |  |  |
|  |  | AGN | 0.91 | 1.00 | 0.95 | 0.999 | 1.00 |  |  |
|  |  | ACC | 0.97 | 1.00 | 0.99 | 1.000 | 1.00 |  |  |
|  | Venous | AA | 0.94 | 0.59 | 0.73 | 0.648 | 0.79 | 0.905 | 0.873 |
|  |  | PCC | 0.96 | 0.90 | 0.93 | 0.917 | 0.95 |  |  |
|  |  | AM | 0.99 | 0.98 | 0.99 | 0.983 | 0.99 |  |  |
|  |  | AC | 0.98 | 0.98 | 0.98 | 0.983 | 0.99 |  |  |
|  |  | AGN | 0.91 | 0.88 | 0.90 | 0.900 | 0.94 |  |  |
|  |  | ACC | 0.86 | 1.00 | 0.92 | 0.999 | 1.00 |  |  |
| Fast R-CNN | Unenhanced | AA | 0.47 | 0.22 | 0.30 | 0.32 | 0.40 | 0.63 | 0.550 |
|  |  | PCC | 0.94 | 0.26 | 0.41 | 0.46 | 0.66 |  |  |
|  |  | AM | 0.96 | 1.00 | 0.99 | 0.98 | 0.98 |  |  |
|  |  | AC | 0.51 | 0.69 | 0.59 | 0.59 | 0.85 |  |  |
|  |  | AGN | 0.80 | 0.51 | 0.62 | 0.63 | 0.77 |  |  |
|  |  | ACC | 0.45 | 0.94 | 0.61 | 0.84 | 0.93 |  |  |
|  | Arterial | AA | 0.98 | 0.40 | 0.57 | 0.50 | 0.80 | 0.88 | 0.770 |
|  |  | PCC | 0.94 | 0.87 | 0.90 | 0.89 | 0.92 |  |  |
|  |  | AM | 1.00 | 1.00 | 1.00 | 1.00 | 1.00 |  |  |
|  |  | AC | 0.79 | 1.00 | 0.88 | 0.98 | 0.98 |  |  |
|  |  | AGN | 0.91 | 0.98 | 0.94 | 0.97 | 0.97 |  |  |
|  |  | ACC | 0.89 | 0.98 | 0.93 | 0.97 | 0.98 |  |  |
|  | Venous | AA | 0.87 | 0.55 | 0.67 | 0.71 | 0.79 | 0.87 | 0.764 |
|  |  | PCC | 0.91 | 0.82 | 0.86 | 0.84 | 0.90 |  |  |
|  |  | AM | 0.92 | 1.00 | 0.96 | 0.94 | 0.97 |  |  |
|  |  | AC | 0.85 | 0.91 | 0.88 | 0.95 | 0.98 |  |  |
|  |  | AGN | 0.93 | 0.87 | 0.90 | 0.89 | 0.90 |  |  |
|  |  | ACC | 0.84 | 0.95 | 0.89 | 0.91 | 0.93 |  |  |
| EfficientDet | Unenhanced | AA | 0.71 | 0.82 | 0.77 | 0.73 | 0.75 | 0.71 | 0.820 |
|  |  | PCC | 0.63 | 0.82 | 0.71 | 0.79 | 0.78 |  |  |
|  |  | AM | 0.95 | 0.98 | 0.96 | 0.97 | 0.97 |  |  |
|  |  | AC | 0.96 | 0.58 | 0.68 | 0.66 | 0.75 |  |  |
|  |  | AGN | 0.83 | 0.59 | 0.69 | 0.66 | 0.77 |  |  |
|  |  | ACC | 0.59 | 0.73 | 0.65 | 0.47 | 0.63 |  |  |
|  | Arterial | AA | 1.00 | 0.47 | 0.63 | 0.54 | 0.72 | 0.86 | 0.840 |
|  |  | PCC | 0.85 | 0.87 | 0.86 | 0.86 | 0.85 |  |  |
|  |  | AM | 0.97 | 0.98 | 0.98 | 0.97 | 0.97 |  |  |
|  |  | AC | 0.73 | 0.95 | 0.83 | 0.94 | 0.98 |  |  |
|  |  | AGN | 0.90 | 0.98 | 0.93 | 0.94 | 0.95 |  |  |
|  |  | ACC | 0.88 | 0.95 | 0.92 | 0.91 | 0.93 |  |  |
|  | Venous | AA | 0.93 | 0.68 | 0.76 | 0.70 | 0.78 | 0.88 | 0.870 |
|  |  | PCC | 0.93 | 0.80 | 0.88 | 0.85 | 0.88 |  |  |
|  |  | AM | 0.98 | 0.98 | 0.99 | 0.98 | 0.98 |  |  |
|  |  | AC | 0.98 | 0.97 | 0.98 | 0.98 | 0.98 |  |  |
|  |  | AGN | 0.94 | 0.88 | 0.90 | 0.91 | 0.92 |  |  |
|  |  |  | 0.82 | 0.98 | 0.89 | 0.90 | 0.92 |  |  |
| MA-YOLO | Unenhanced | AA | 0.91 | 0.66 | 0.77 | 0.696 | 0.83 | 0.885 | 0.838 |
|  |  | PCC | 0.88 | 0.89 | 0.89 | 0.896 | 0.94 |  |  |
|  |  | AM | 1.00 | 0.98 | 0.99 | 0.988 | 0.99 |  |  |
|  |  | AC | 0.92 | 0.81 | 0.86 | 0.842 | 0.90 |  |  |
|  |  | AGN | 0.85 | 0.94 | 0.89 | 0.921 | 0.96 |  |  |
|  |  | ACC | 0.88 | 0.99 | 0.93 | 0.968 | 0.99 |  |  |
|  | Arterial | AA | 1.00 | 0.49 | 0.65 | 0.564 | 0.74 | 0.913 | 0.885 |
|  |  | PCC | 0.94 | 0.90 | 0.92 | 0.915 | 0.95 |  |  |
|  |  | AM | 1.00 | 1.00 | 1.00 | 1.000 | 1.00 |  |  |
|  |  | AC | 0.89 | 1.00 | 0.94 | 0.997 | 1.00 |  |  |
|  |  | AGN | 0.91 | 1.00 | 0.95 | 1.000 | 1.00 |  |  |
|  |  | ACC | 0.91 | 1.00 | 0.95 | 0.999 | 1.00 |  |  |
|  | Venous | AA | 1.00 | 0.66 | 0.80 | 0.714 | 0.83 | 0.915 | 0.890 |
|  |  | PCC | 0.97 | 0.84 | 0.90 | 0.873 | 0.92 |  |  |
|  |  | AM | 1.00 | 0.98 | 0.99 | 0.987 | 0.99 |  |  |
|  |  | AC | 0.98 | 1.00 | 0.99 | 1.000 | 1.00 |  |  |
|  |  | AGN | 0.88 | 0.99 | 0.94 | 0.939 | 0.99 |  |  |
|  |  | ACC | 0.85 | 0.98 | 0.91 | 0.977 | 0.99 |  |  |

**4. Reference**

1. Hu J, Shen L, Albanie S, Sun G, Wu E. Squeeze-and-Excitation Networks. IEEE Trans Pattern Anal Mach Intell. 2020 Aug;42(8):2011-23. PMID: 31034408. doi: 10.1109/tpami.2019.2913372.

2. Woo S, Park J, Lee J-Y, Kweon IS, editors. Cbam: Convolutional block attention module. Proceedings of the European conference on computer vision (ECCV); 2018.
