# Supplementary material for: Localization and Classification of Adrenal Masses in Multiphase Computed Tomography: Retrospective Study
Source: J Med Internet Res. 2025 Apr 24;27:e65937. doi: 10.2196/65937 (PMC12062765; doi:10.2196/65937)
Supplement: Multimedia Appendix 2 [file jmir_v27i1e65937_app2.docx]

## Multimedia Appendix 2

**Table S3.** Comparison of performance for each subclass of adrenal masses between independent diagnosis by physicians and by the model.

|  | Model (AUC^a^) | Junior clinician | | | Intermediate clinician | | | Senior clinician | | | Junior radiologist | | | Intermediate radiologist | | | Senior radiologist | | |
| --- | --- | --- | --- | --- | --- | --- | --- | --- | --- | --- | --- | --- | --- | --- | --- | --- | --- | --- | --- |
|  |  | AUC | *z* value^b^ | *P* value | AUC | *z* value^b^ | *P* value | AUC | *z* value^b^ | *P* value | AUC | *z* value^b^ | *P* value | AUC | *z* value^b^ | *P* value | AUC | *z* value^b^ | *P* value |
|  |  |  |  |  |  |  |  |  |  |  |  |  |  |  |  |  |  |  |  |
| AA^c^ | 0.83 | 0.75 | 1.133 | .26 | 0.75 | 1.678 | .09 | 0.89 | –0.714 | .48 | 0.59 | 3.066 | .00 | 0.81 | 0.532 | .60 | 0.71 | 4.149 | <.001 |
| PCC^d^ | 0.92 | 0.63 | 2.298 | .02 | 0.71 | 2.263 | .02 | 0.69 | 1.794 | .07 | 0.61 | 2.091 | .04 | 0.67 | 2.617 | .01 | 0.59 | 3.487 | <.001 |
| AM^e^ | 0.99 | 0.85 | 1.818 | .07 | 0.86 | 1.850 | .06 | 0.98 | 0.573 | .57 | 0.96 | 1.769 | .08 | 0.98 | 0.573 | .57 | 0.9 | 1.335 | .18 |
| AC^f^ | 1.00 | 0.74 | 2.413 | .02 | 0.80 | 1.818 | .07 | 0.84 | 1.467 | .14 | 0.65 | 3.525 | <.001 | 0.8 | 1.975 | .05 | 0.83 | 1.596 | .11 |
| AGN^g^ | 0.99 | 0.77 | 1.177 | .24 | 0.64 | 2.202 | .03 | 0.68 | 1.876 | .06 | 0.54 | 4.504 | <.001 | 0.45 | 6.208 | <.001 | 0.66 | 2.718 | .01 |
| ACC^h^ | 0.99 | 0.82 | 2.059 | .04 | 0.80 | 2.086 | .04 | 0.9 | 1.217 | .22 | 0.61 | 4.450 | <.001 | 0.67 | 3.516 | <.001 | 0.69 | 3.331 | <.001 |

^a^AUC: area under the receiver operating characteristic curve.

^b^The *z* value is the test statistic in the DeLong test.

^c^AA: adrenocortical adenoma.

^d^PCC: pheochromocytoma.

^e^AM: adrenal myelolipoma.

^f^AC: adrenal cyst.

^g^AGN: adrenal ganglioneuroma.

^h^ACC: adrenocortical carcinoma.
