# Supplementary material for: Localization and Classification of Adrenal Masses in Multiphase Computed Tomography: Retrospective Study
Source: J Med Internet Res. 2025 Apr 24;27:e65937. doi: 10.2196/65937 (PMC12062765; doi:10.2196/65937)
Supplement: Multimedia Appendix 3 [file jmir_v27i1e65937_app3.docx]

## Multimedia Appendix 3

**Table S4.** Comparison of diagnostic performance for each subclass of adrenal masses between independent diagnoses by physicians or with assistance from the Multi-Attention YOLO (MA-YOLO) model.

|  | Junior clinician (AUC^a^) | Junior clinician + MA-YOLO assisted | | | Intermediate clinician (AUC) | Intermediate clinician + MA-YOLO assisted | | | Senior clinician (AUC) | Senior clinician + MA-YOLO assisted | | | Junior radiologist (AUC) | Junior radiologist + MA-YOLO assisted | | | Intermediate radiologist (AUC) | Intermediate radiologist + MA-YOLO assisted | | | Senior radiologist (AUC) | Senior radiologist + MA-YOLO assisted | | |
| --- | --- | --- | --- | --- | --- | --- | --- | --- | --- | --- | --- | --- | --- | --- | --- | --- | --- | --- | --- | --- | --- | --- | --- | --- |
|  |  | AUC | *z* value ^b^ | *P* value |  | AUC | *z* value ^b^ | *P* value |  | AUC | *z* value ^b^ | *P* value |  | AUC | *z* value ^b^ | *P* value |  | AUC | *z* value ^b^ | *P* value |  | AUC | *z* value ^b^ | *P* value |
|  |  |  |  |  |  |  |  |  |  |  |  |  |  |  |  |  |  |  |  |  |  |  |  |  |
| AA^c^ | 0.75 | 0.84 | –1.622 | .11 | 0.75 | 0.83 | –1.515 | .13 | 0.89 | 0.93 | –2.064 | .04 | 0.59 | 0.81 | –2.697 | .01 | 0.81 | 0.8 | 0.131 | .90 | 0.71 | 0.78 | –2.824 | .01 |
| PCC^d^ | 0.63 | 0.79 | –2.077 | .04 | 0.71 | 0.82 | –1.596 | .11 | 0.69 | 0.84 | –1.964 | .05 | 0.61 | 0.78 | –1.619 | .11 | 0.67 | 0.75 | –1.515 | .13 | 0.59 | 0.82 | –2.758 | .01 |
| AM^e^ | 0.85 | 0.94 | –1.335 | .18 | 0.86 | 0.99 | –1.850 | .06 | 0.98 | 0.99 | –0.573 | .57 | 0.96 | 0.98 | –1.000 | .32 | 0.98 | 0.99 | –1.000 | .32 | 0.9 | 0.95 | –1.000 | .32 |
| AC^f^ | 0.74 | 0.84 | –0.990 | .32 | 0.80 | 0.95 | –1.964 | .05 | 0.84 | 0.94 | –1.500 | .13 | 0.65 | 0.95 | –3.674 | <.001 | 0.8 | 0.94 | –2.017 | .04 | 0.83 | 0.9 | –1.348 | .18 |
| AGN^g^ | 0.77 | 0.76 | 0.131 | .90 | 0.64 | 0.8 | –1.480 | .14 | 0.68 | 0.84 | –2.077 | .04 | 0.54 | 0.75 | –2.553 | .01 | 0.45 | 0.51 | –1.133 | .26 | 0.66 | 0.81 | –1.930 | .05 |
| ACC^h^ | 0.82 | 0.87 | –2.333 | .02 | 0.80 | 0.83 | –1.353 | .18 | 0.9 | 0.98 | –1.463 | .14 | 0.61 | 0.83 | –2.656 | .01 | 0.67 | 0.93 | –3.098 | .001 | 0.69 | 0.84 | –1.930 | .05 |

^a^AUC: area under the receiver operating characteristic curve.

^b^The Z value is the test statistic in the DeLong test.

^c^AA: adrenocortical adenoma.

^d^PCC: pheochromocytoma.

^e^AM: adrenal myelolipoma.

^f^AC: adrenal cyst.

^g^AGN: adrenal ganglioneuroma.

^h^ACC: adrenocortical carcinoma.
